# Time course of pulmonary inflammation and trace element biodistribution during and after sub-acute inhalation exposure to copper oxide nanoparticles in a murine model

**DOI:** 10.1186/s12989-022-00480-z

**Published:** 2022-06-13

**Authors:** Sudartip Areecheewakul, Andrea Adamcakova-Dodd, Ezazul Haque, Xuefang Jing, David K. Meyerholz, Patrick T. O’Shaughnessy, Peter S. Thorne, Aliasger K. Salem

**Affiliations:** 1 Department of Pharmaceutical Sciences and Experimental Therapeutics, The University of Iowa, College of Pharmacy, Iowa City, IA 52242 USA; 2 Department of Occupational and Environmental Health, The University of Iowa, College of Public Health, Iowa City, IA 52242 USA; 3 Interdisciplinary Graduate Program in Human Toxicology, University of Iowa, Iowa City, IA 52246 USA; 4 Department of Pathology, University of Iowa, Iowa City, IA 52242 USA

**Keywords:** Copper oxide nanoparticles (CuO NPs), Nanomaterial, Pulmonary toxicity, Inhalation, Cytokines, Inflammation, Trace elements

## Abstract

**Background:**

It has been shown that copper oxide nanoparticles (CuO NPs) induce pulmonary toxicity after acute or sub-acute inhalation exposures. However, little is known about the biodistribution and elimination kinetics of inhaled CuO NPs from the respiratory tract. The purposes of this study were to observe the kinetics of pulmonary inflammation during and after CuO NP sub-acute inhalation exposure and to investigate copper (Cu) biodistribution and clearance rate from the exposure site and homeostasis of selected trace elements in secondary organs of BALB/c mice.

**Results:**

Sub-acute inhalation exposure to CuO NPs led to pulmonary inflammation represented by increases in lactate dehydrogenase, total cell counts, neutrophils, macrophages, inflammatory cytokines, iron levels in bronchoalveolar lavage (BAL) fluid, and lung weight changes. Dosimetry analysis in lung tissues and BAL fluid showed Cu concentration increased steadily during exposure and gradually declined after exposure. Cu elimination from the lung showed first-order kinetics with a half-life of 6.5 days. Total Cu levels were significantly increased in whole blood and heart indicating that inhaled Cu could be translocated into the bloodstream and heart tissue, and potentially have adverse effects on the kidneys and spleen as there were significant changes in the weights of these organs; increase in the kidneys and decrease in the spleen. Furthermore, concentrations of selenium in kidneys and iron in spleen were decreased, pointing to disruption of trace element homeostasis.

**Conclusions:**

Sub-acute inhalation exposure of CuO NPs induced pulmonary inflammation, which was correlated to Cu concentrations in the lungs and started to resolve once exposure ended. Dosimetry analysis showed that Cu in the lungs was translocated into the bloodstream and heart tissue. Secondary organs affected by CuO NPs exposure were kidneys and spleen as they showed the disruption of trace element homeostasis and organ weight changes.

**Supplementary Information:**

The online version contains supplementary material available at 10.1186/s12989-022-00480-z.

## Background

Nanomaterials have been widely used in diverse fields due to their unique physicochemical properties including high surface-to-volume ratios, quantum effects, and flexible functionalization of surface structures [1, 2]. Copper oxide nanoparticles (CuO NPs) have been used in several commercial applications such as antimicrobial agents, solar cells, catalysts, and electronic products [3–6]. Copper (Cu) is an essential transition metal element for most organisms, from bacteria to humans. A variety of cellular processes require Cu as a redox-reactive substance and as cofactors in enzymatic reactions involving oxidative phosphorylation, iron (Fe) uptake and mobilization, oxygen transport, antioxidative defense, neuropeptide maturation, blood clotting, and angiogenesis [7–9]. However, excess Cu leads to reversible redox changes and potentially causes deleterious effects. Excess Cu can generate high levels of reactive oxygen species (ROS) by cells due to lipid peroxidation in the cell membrane, oxidation of protein, and cleavage of DNA molecules [10]. Imbalance of Cu homeostasis results in certain serious disorders such as Menkes disease and Wilson’s disease, which are human genetic diseases affecting Cu transport [8]. Furthermore, trace elements can exert a vast influence on one another. For instance, high intakes of Cu, zinc, or cadmium can interfere with tissue storage and utilization of Fe [10].

With increasing applications for the use of CuO NP and the resulting concern their potentially harmful effects, nanomaterial toxicity research both in vitro and in vivo has dramatically increased [11, 12]. Previous work by our group focused on assessing the toxicity of a range of nanomaterials including ZnO, V_2_O_5_, WO_3_, Al_2_O_3_, Fe_2_O_3_, TiO_2_, MgO, and CeO_2_, and revealed CuO NPs to exert the greatest cytotoxicity in a dose-dependent manner [13]. In vitro studies have been performed to determine the toxicity mechanism at a molecular level. However, one of the limitations of in vitro toxicity assessment is that it may not reflect accurately outcomes under in vivo conditions. Moreover, in vitro testing doses typically exceed an environmentally exposed human dose. In vivo studies were therefore applied in this study to better characterize overall toxicity and trace elemental homeostasis after nanoparticulate inhalation. NP production and processing are mostly performed in contained systems; however, ultrafine NPs may escape containment and diffuse into the environment and be inhaled leading to potential harmful effects, especially to workers [14, 15]. Moreover, inhalation is the most significant route by which people have been unintentionally exposed to nanomaterials [16]. Therefore, scientists and the public have been widely concerned about inhalation exposure of CuO NPs. Inhalation exposure to CuO NPs can induce pulmonary inflammation, increase levels of inflammatory cells, proinflammatory cytokines, ROS, and/or airway hyperresponsiveness in a murine model [17–20]. We previously examined [19] the pulmonary inflammatory response of sub-acutely exposed (4 h per day, 5 days per week for 2 weeks) mice by whole-body inhalation of Cu NPs at 3.6 mg/m^3^. Immediately after exposure, mice had significantly higher levels of bronchoalveolar lavage (BAL) cytokines, inflammatory cells, lactate dehydrogenase (LDH), as well as perivasculitis and alveolitis. At 3-weeks post-exposure, all toxicity parameters and histopathology returned to typical values except the number of inflammatory cells. The histopathology of the lung at 0- and 3-weeks post-exposure demonstrated the absence of Cu NPs, suggesting the possibility of Cu NPs translocation from the pulmonary site to other organs or Cu dissolution in the airways or airway surface liquid. Moreover, in an ovalbumin (OVA)-induced asthma mouse model, CuO NPs aggravated the increased airway hyperresponsiveness, inflammatory cell count, proinflammatory cytokines, reactive oxidation species, and immunoglobulin E induced by OVA exposure [18]. Similarly, rats exposed to CuO NP aerosols for 5 days (6 h/day) showed dose-dependent lung inflammation and cytotoxicity indicated by increases in LDH, total cells and neutrophils in BAL fluid, changes in lung histopathology as showing hyperplasia of bronchiolar and alveolar epithelium, interstitial, and alveolar inflammation at 24-h post-exposure. The effects were almost completely resolved during a 3-week post exposure [17, 20]. Studies on translocation, biodistribution and fate of nanomaterials could help predict systemic adverse effects on primary organ exposure and other secondary organs [21]. However, in vivo studies of NP translocation to secondary organs and biodistribution data are scarce and therefore this topic is not well understood. To better understand the accumulation and clearance of NPs at the exposure site and time-dependent biological changes induced by NPs, a study was needed with assessment of adverse effects at multiple time points during the exposure and recovery period after exposure [22].

This study aimed to investigate the kinetics of pulmonary inflammation and biodistribution of inhaled CuO NPs during and after subacute exposure. Tracking Cu concentration in the lungs over time would deepen our understanding of CuO NP toxicity at the exposed site. Moreover, fluctuations in Cu and other trace element concentrations in secondary organs during and after exposure may lead to disruptions in trace element homeostasis and subsequently to systemic adverse effects since trace elements have essential roles in biochemical processes in the body and are heavily dependent on one another.

In this study, female BALB/c mice were exposed to CuO NPs at 3.75 mg/m^3^ using a nose-only exposure system for 4 h/day, 5 days/week over 2 weeks, and were necropsied on days 0, 3, 7, 12, 17, 22, and 27 (Day 3, 7, and 12 were during exposure while days 17, 22, and 27 were post exposure). BAL fluids were collected to measure inflammatory cytokine/chemokines levels, numbers of white blood cells, and LDH. Lung histopathology was performed to evaluate inflammation. Whole blood, lung, brain, heart, kidneys, liver, spleen, urine, and BAL fluid were collected to measure Cu and selected trace element concentrations. Disruption of the elemental homeostasis in primary and secondary organs might be an indication of negative adverse effects in these biological systems. To better understand how CuO NPs are processed and mobilized once they are inhaled, dissolution studies in three artificial biological fluids including artificial lysosomal fluid (ALF, pH 4.5), simulated epithelial lung fluid (SELF, pH 7.4), and simulated gastric fluid (SGF, pH 1.5) were conducted.

## Results

### CuO NP characterization and particle size distribution of CuO NP aerosol during exposure

**Fig. 1:**
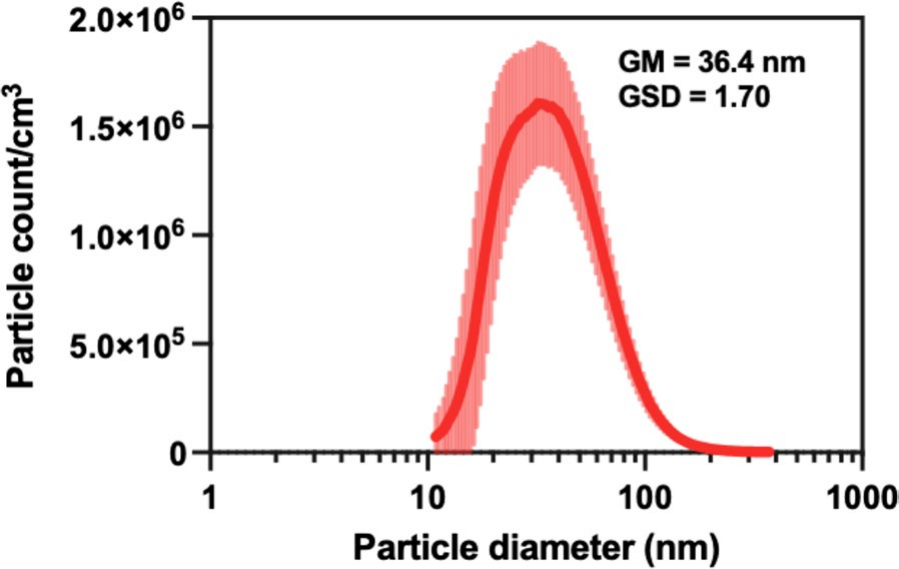
Particle size distribution of CuO NP aerosols measured by a scanning mobility particle sizer (SMPS) from the nose-only port of the exposure system during inhalation exposure. GM = geometric mean, GSD = geometric standard deviation

The CuO NPs and characterization data were provided by the Engineered Nanomaterials Resource and Coordination Core (ERCC) (Table 1). CuO NP density measured by pycnometer (N_2_ volume displacement) was 6.15 ± 0.0027 g/cm^3^. During inhalation exposure, the particle size distribution of CuO NP aerosol measured by a scanning mobility particles sizer (SMPS) spectrometer showed a geometric mean (GM) mobility diameter of 36.4 nm with a geometric standard deviation (GSD) of 1.67 (Fig. 1). Similarly, our previous acute inhalation study using the same material showed that CuO NP aerosols were generated with GM of 33.3 nm and GSD of 1.70 [13]. The CuO NP aerosol concentration was 3.75 ± 0.26 mg/m^3^, which was close to our target concentration of 3.5 mg/m^3^.

**Table 1:** Summary of CuO NP characterization from ERCC

Characterization parameters	Methods	CuO
*Primary particle size*		
d_XRD_* (nm) Specific surface area (SSA) (m^2^/g) d_BET#_ (nm) Mean size (nm)	X-ray diffraction Brunauer-Emmett teller (BET) BET TEM	25.8 13.77 ± 0.68 70.9 ± 3.54 50.24 ± 10.99
Hydrodynamic size in DI water (nm)	Dynamic light scattering	507 ± 51
Zeta potential in DI water (mV)	Dynamic light scattering	-19.0 ± 1.5
*Shape factors*		
Aspect ratio ξ Circularity ψ Roundness ζ	TEM TEM TEM	1.345 ± 0.202 0.925 ± 0.037 0.759 ± 0.107
*Porosity*		
Pore volume (cm^3^/g) Average pore size (nm)	BET BET	1.87 × 10^–4^ 2.65
Density (g/cm^3^)	Pycnometer (N_2_ volume replacement)	6.1531 ± 0.0027
Purity	Inductively coupled plasma mass spectrometry (ICP-MS)	98.21% ± 6.11% Cu
*Endotoxin levels*		
Concentration in suspension (EU/mL) Concentration per mass of ENM (EU/mg)	Recombinant factor C Recombinant factor C	0.021 2.141

### Pulmonary toxicity assessment during and after sub-acute CuO NP inhalation exposure

**Fig. 2:**
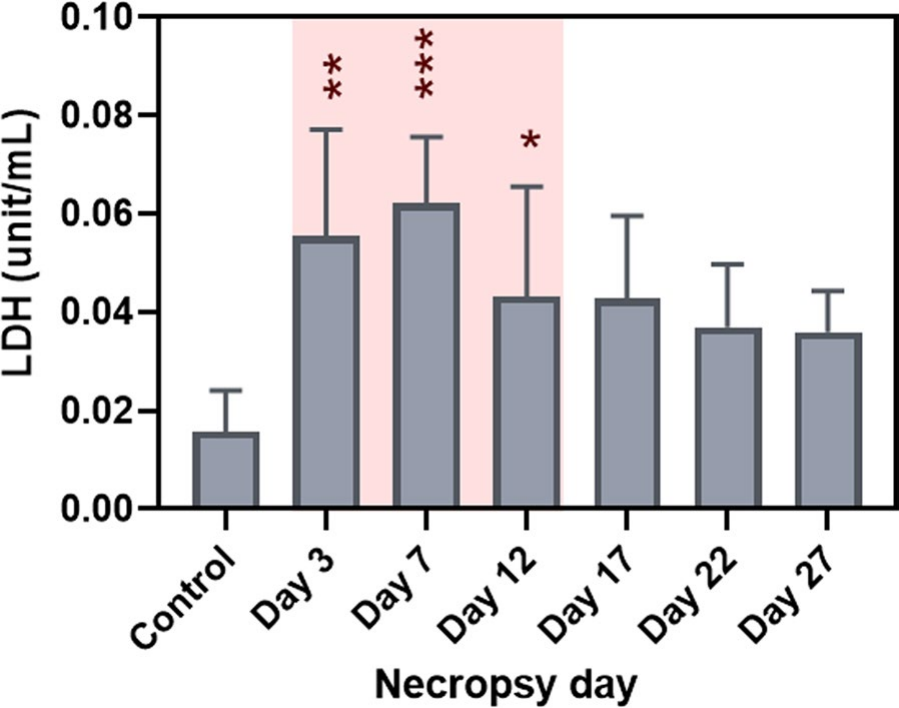
Lactate dehydrogenase enzyme levels in BAL fluid (Red-highlighted area indicated time during CuO exposure). Statistical analysis was performed using one-way ANOVA with Dunnett’s post hoc test. Data are expressed as mean ± SD (n = 5). *** P < 0.001, **P < 0.01, *P < 0.05

**Fig. 3:**
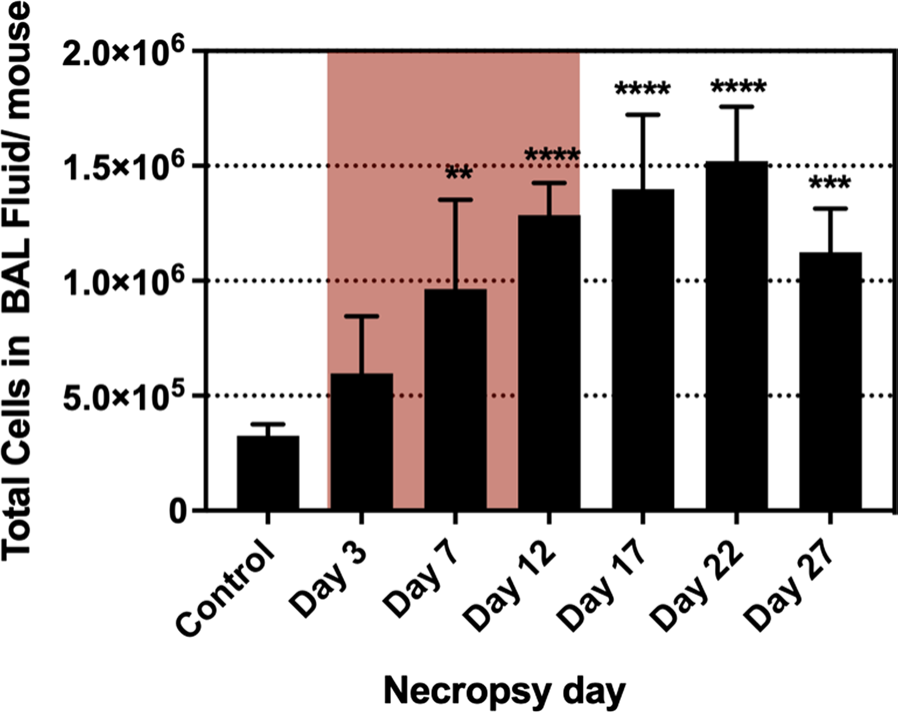
Total cell count in BAL fluid (Red-highlighted area indicated time during CuO exposure). Statistical analysis was performed using one-way ANOVA with Dunnett’s post hoc test. Data are expressed as mean ± SD (n = 5). ****P < 0.0001, *** P < 0.001, **P < 0.01

**Fig. 4:**
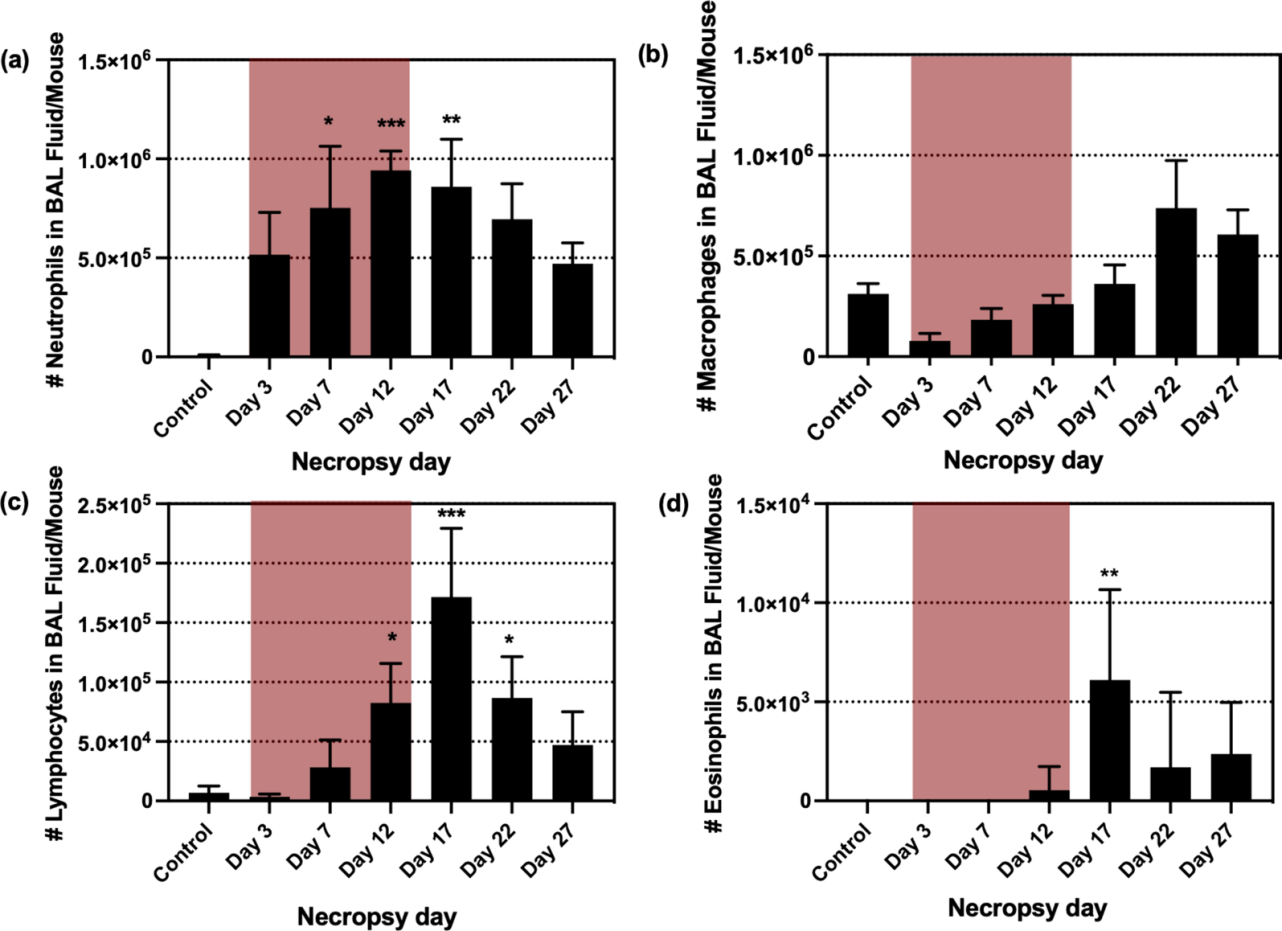
Inflammatory cell numbers in BAL fluid of mice exposed to CuO NPs in aerosols. **a** Neutrophils, **b** Macrophages, **c** Lymphocytes, and **d** Eosinophils (Red-highlighted area indicated time during CuO exposure). Statistical analysis was performed using Kruskal–Wallis test. Data are expressed as mean ± SD (n = 5). *** P < 0.001, **P < 0.01, *P < 0.05

**Fig. 5:**
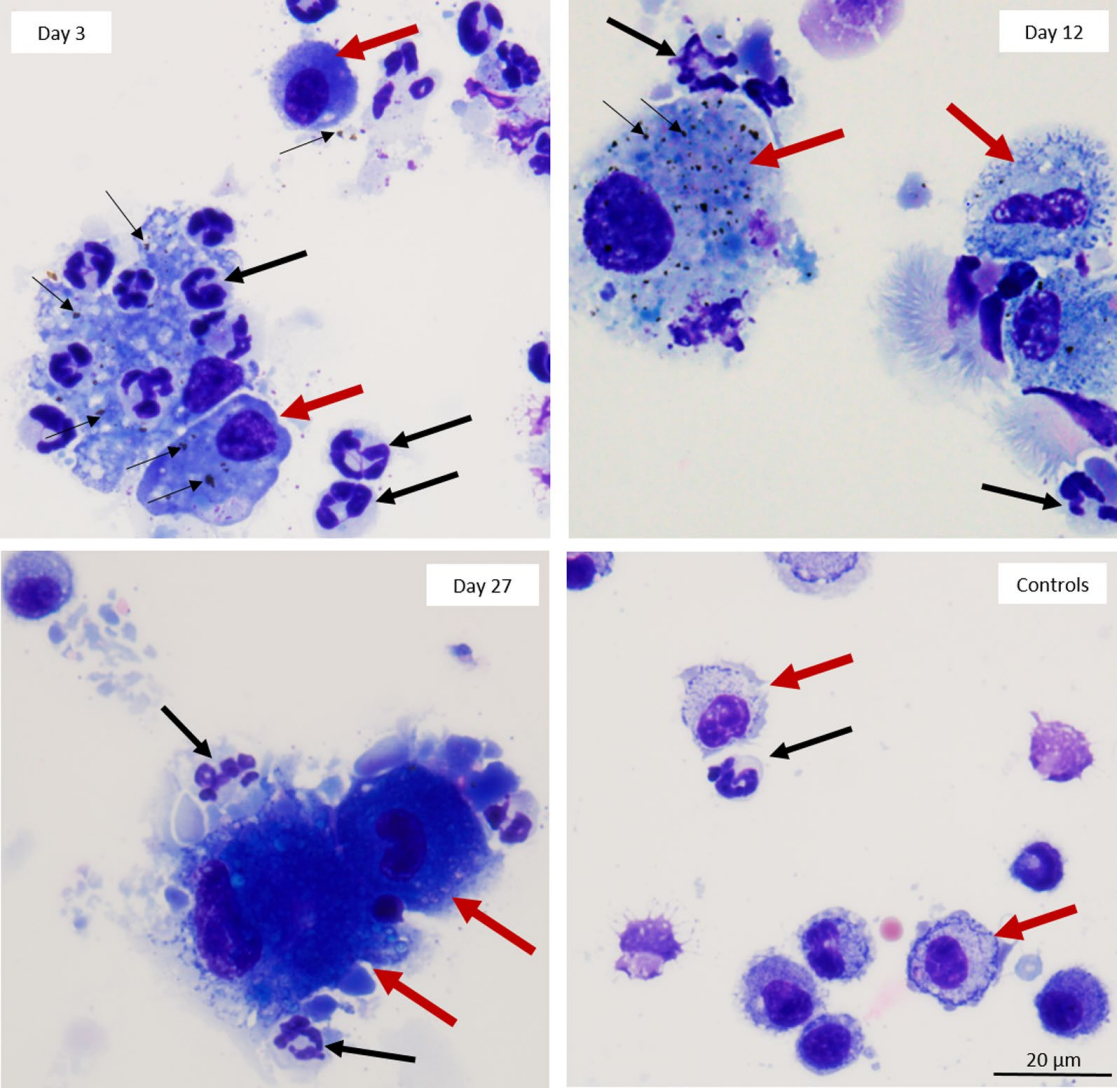
Micrographs of leukocytes in BAL fluid from mice exposed to CuO NPs and necropsied on Days 3, 12 and 27 and control mice. Aggregated CuO NPs are visible on Day 3 inside the macrophages as well as free between the cells (thin black arrows). Recruitment of neutrophils is present on Day 3–Day 27 (thick black arrows). The appearance of macrophages (thick red arrows) changes overtime; becoming progressively more activated and enlarged from Day 3 to Day 27. Cells were stained with Protocol® HEMA 3 stain set

LDH levels increased for all time points compared to the control group, however, significant increases were observed on day 3 (p < 0.01), 7 (p < 0.001), and 12 (p < 0.05) which were the days of the exposure period (Fig. 2). The total cells from BAL fluid were significantly increased after day 7 (p < 0.01), and the highest cell number was on day 22 (p < 0.0001), and then decreased by day 27, but was still higher than control group (p < 0.001) (Fig. 3). The increases in total cell count from BAL fluid were mainly due to elevation in neutrophils and macrophages (Fig. 4a, b). Neutrophil numbers were increased relative to the control at all time points and started declining after the end of exposure (with significances on day 7 (p < 0.05), day 12 (p < 0.001), and day 17 (p < 0.01) (Fig. 4a). The number of macrophages initially declined (compared to control), albeit not significantly, during exposure and gradually increased (not significantly) including after exposure period (Fig. 4b). Lymphocytes showed significant increases on day 12 (p < 0.05), 17 (p < 0.001), and 22 (p < 0.05) (Fig. 4c), although the numbers were proportionally low when compared to neutrophils and macrophages. The numbers of eosinophils in the BAL fluid were significantly increased only on day 17 (p < 0.01), but they were very low throughout the study (Fig. 4d). There were only 0–1.0% of eosinophils in BAL out of the total differential cells in the exposed mice. The percentage of neutrophils, macrophages and lymphocytes in exposed mice were as follows: 31.0–89.8%, 9.5–62.0%, and 0–11.0%, respectively, as shown in Additional file 1: Figure S1. As inspected by light microscopy, the macrophages from exposed animals became more activated over time and enlarged with foamy vacuoles compared to controls (Fig. 5, selected time points are shown). Aggregated/agglomerated particulates engulfed in the macrophages were more visible at earlier time points (day 3–12). At later time points (after day 12), the vacuoles of macrophages were more foamy and particles were less noticeable as shown on the micrograph from day 27, suggesting conceivable transformation of particulates (e.g. particle dissolution). Moreover, a multiple linear regression was calculated to predict the number of each type of leukocyte in BAL fluid (as a dependent variable) based on Cu concentration in the lung tissue and time (as independent variables). We found that Cu concentration in the lung was a significant predictor of the number of neutrophils, with a positive association (p < 0.0001 with R^2^ = 0.6243), while Cu concentrations were negatively associated with macrophage numbers (p = 0.0013 with R^2^ = 0.6785) when statistically adjusted for time.

**Fig. 6:**
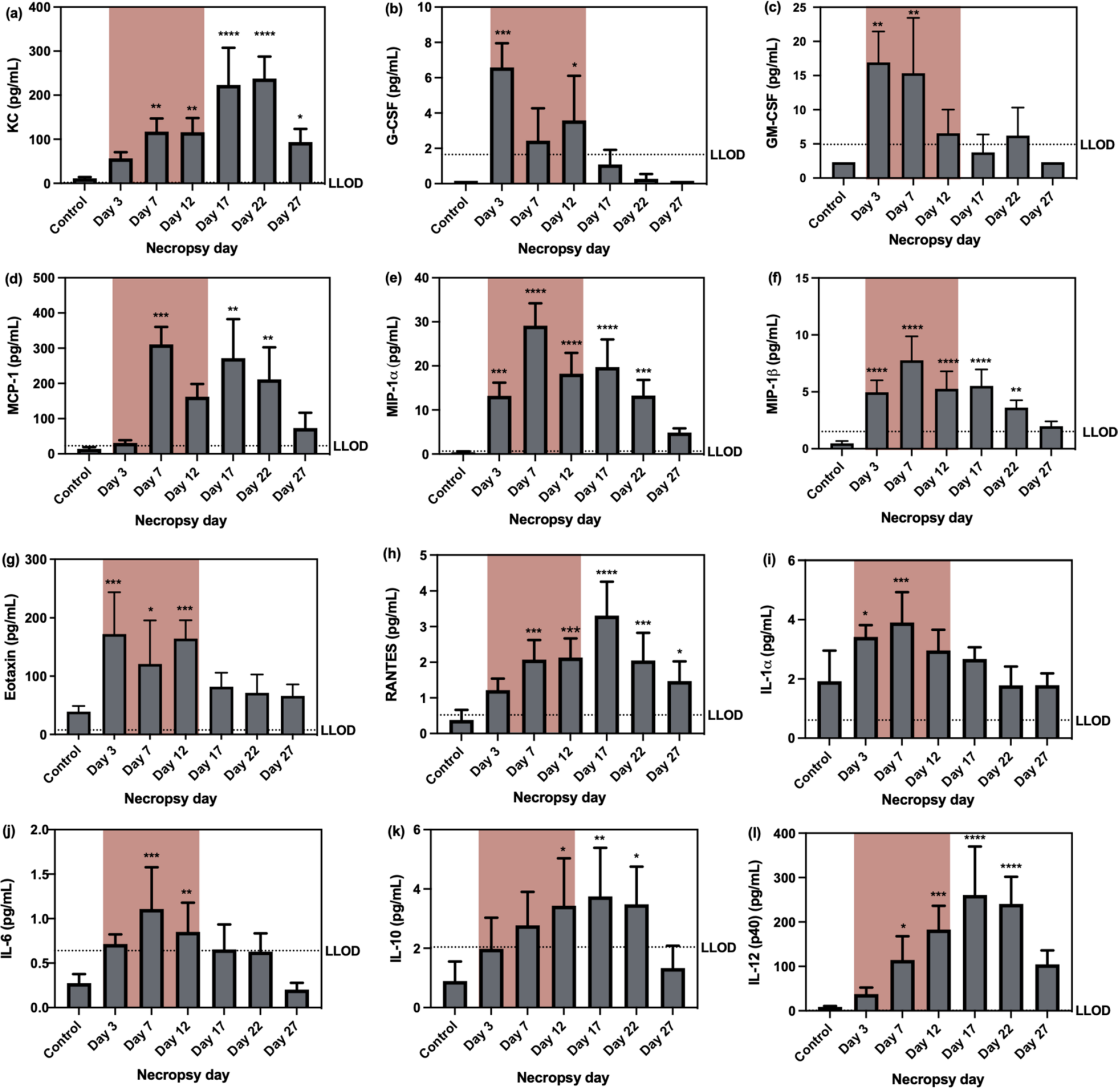
Levels of inflammatory cytokines/chemokines: **a** KC, **b** G-CSF, **c** GM-CSF, **d** MCP-1, **e** MIP-1α, **f** MIP-1β, **g** eotaxin, **h** RANTES, **i** IL-1α, **j** IL-6, **k** IL-10, and **l** IL-12(Pp0) during (red-highlighted area) and after exposure. Statistical analysis of results for KC, MIP-1α, MIP-1β, eotaxin, RANTES, IL-1α, IL-6, IL-10, and IL-12(p40) was performed using one-way ANOVA with Dunnett’s post hoc test, while statistical analysis of results for G-CSF, GM-CSF, and MCP was performed by Kruskal–Wallis test (compare to the control group). Data are expressed as mean ± SD (n = 5). ****P < 0.0001, *** P < 0.001, **P < 0.01, *P < 0.05

Twelve out of 23 cytokines/chemokines measured in BAL fluid showed significant changes during and after exposure and included: keratinocyte chemoattractant (KC), granulocyte colony-stimulating factor (G-CSF), granulocyte–macrophage colony-stimulating factor (GM-CSF), monocyte chemotactic protein (MCP)-1, macrophage inflammatory protein (MIP)-1α, MIP-1β, eotaxin, RANTES, IL-1α, IL-6, IL-10, and IL-12(p40). The concentration of the following cytokines/chemokines was below the lowest limit of detection (LLOD): IL-1β, IL-2, IL-3, IL-4, IL-5, IL-9, IL-12 (p70), IL-17a. No significant changes were found in interferon (IFN)-γ, tumor necrosis factor (TNF)-α, and IL-13 concentrations. Murine KC is a potent chemoattractant, inducing the infiltration of neutrophils to inflammatory sites [23]. The significant increases of KC started at day 7 (p < 0.01) and were further elevated with the maximum level on day 22 (p < 0.0001) and then decreased by day 27, but still higher than the control (p < 0.05) (Fig. 6a). G-CSF production was previously demonstrated to promote the production, maturation, mobilization of granulocytic cells, mainly neutrophils, from bone marrow to sites of injury [23–25]. G-CSF concentrations showed significant elevation on day 3 (p < 0.001) and 12 (p < 0.05) (Fig. 6b). GM-CSF is vital to the development and maintenance of alveolar macrophages [26]. It also recruits circulating of neutrophils, monocytes, and lymphocytes to increase host defense functions [27] and in these studies showed a significant increase on days 3 and 7 (p < 0.01) (Fig. 6c). MCP-1, MIP-1α, and MIP-1β are chemoattractants for monocytes and macrophages, showed a similar increasing trend with maximum levels being reached on day 7 (Fig. 6d–f). Eotaxin and RANTES function as chemoattractants for eosinophils [28]. Eotaxin increased during CuO NP exposure, while RANTES showed significant increases at all time points except day 3 (Fig. 6g, h). IL-1α and IL-6 are cytokines associated with damage to airway epithelial cells, and they showed an increasing trend during exposure (Fig. 6i, j). Conversely, IL-10 and IL-12(p40) subunit are anti-inflammatory cytokines that mitigate the inflammatory responses in the lung [29]. These cytokines showed a similar increasing trend after exposure (Fig. 6k, l).

### Lung histopathology

**Fig. 7:**
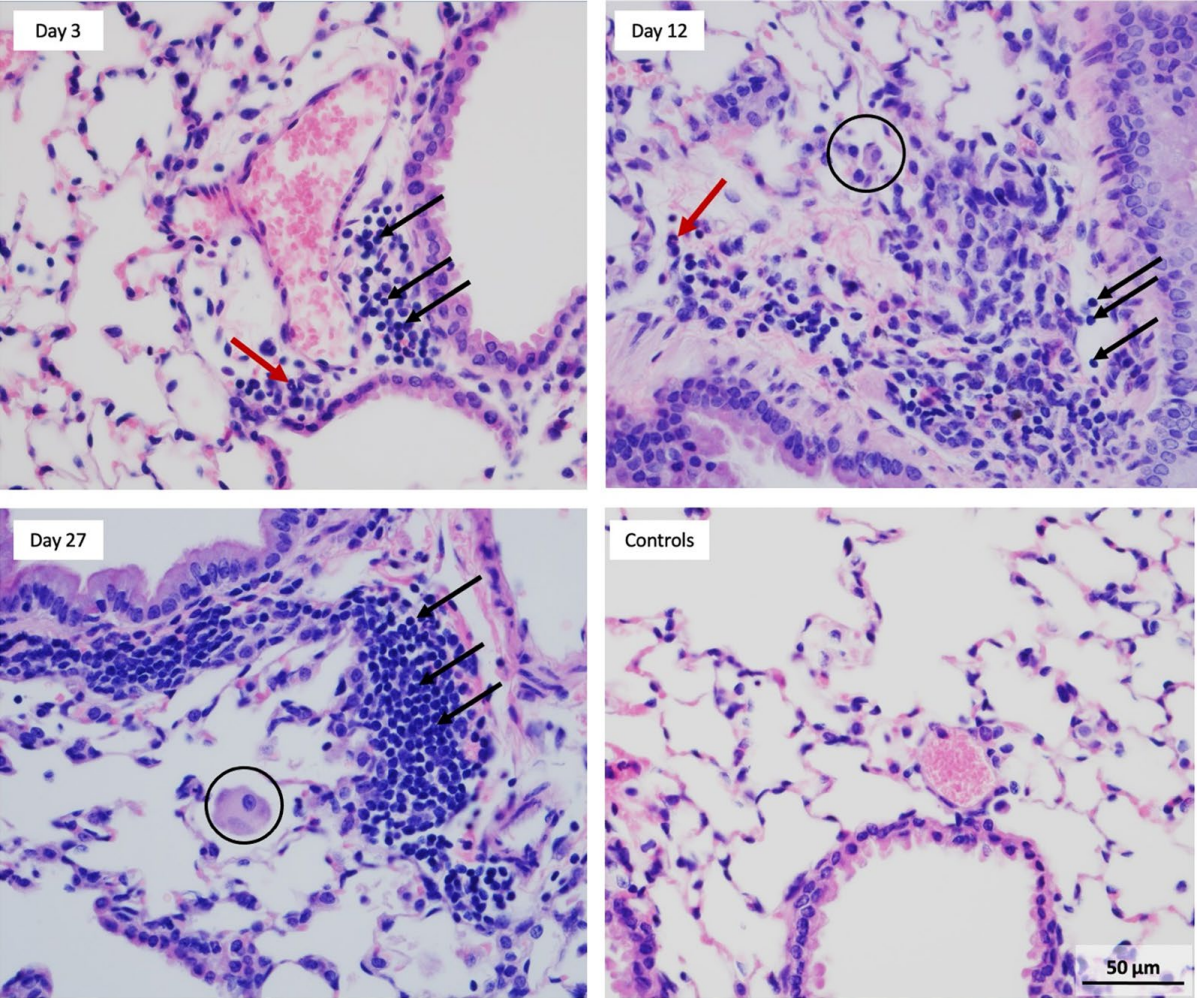
Micrographs of lung sections stained with H&E. Development of progressive perivascular aggregates (black arrows), mainly lymphocytes, indicating chronic inflammation was observed from Day 3 – 27. Occasional neutrophils (red arrows) were seen mainly at earlier time points (Day 3 and Day 12) indicating an acute inflammation. Control mice showed no lesions with clear alveolar spaces and no overt recruitment of lymphocytes or neutrophils. Similarly, as seen in BAL fluid, macrophages became more enlarged and activated with foamy vacuoles (black circles). All images were taken at the same magnification

Micrographs of lung tissues stained with hematoxylin and eosin (H&E) were observed under a bright-field microscope. The controls showed no pathology with clear alveolar spaces and no overt recruitment of lymphocytes or neutrophils. Development of progressive perivascular and periairway inflammation represented mainly by lymphocytes (indicating a chronic inflammation) was observed in the lungs over time starting at day 3 of exposure with increased signs of inflammation present at Day 27 (Fig. 7). Occasional neutrophils at the sites of inflammation were present mainly at earlier time points (Day 3 and 12).

### Trace element distribution in whole blood, organs, and urine

**Fig. 8:**
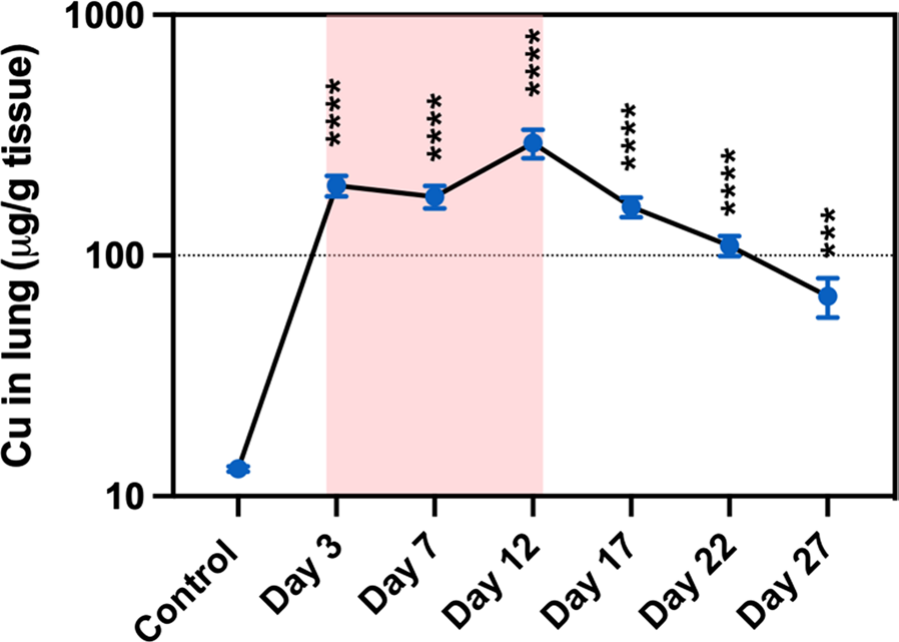
The concentration versus time profile of Cu in lung tissue. Log scale during (red-highlighted area) and after exposure. Statistical analysis was performed using one-way ANOVA with Dunnett’s post hoc test (compared to the control group). Data are expressed as mean ± SD (n = 5). ****P < 0.0001, *** P < 0.001

**Fig. 9:**
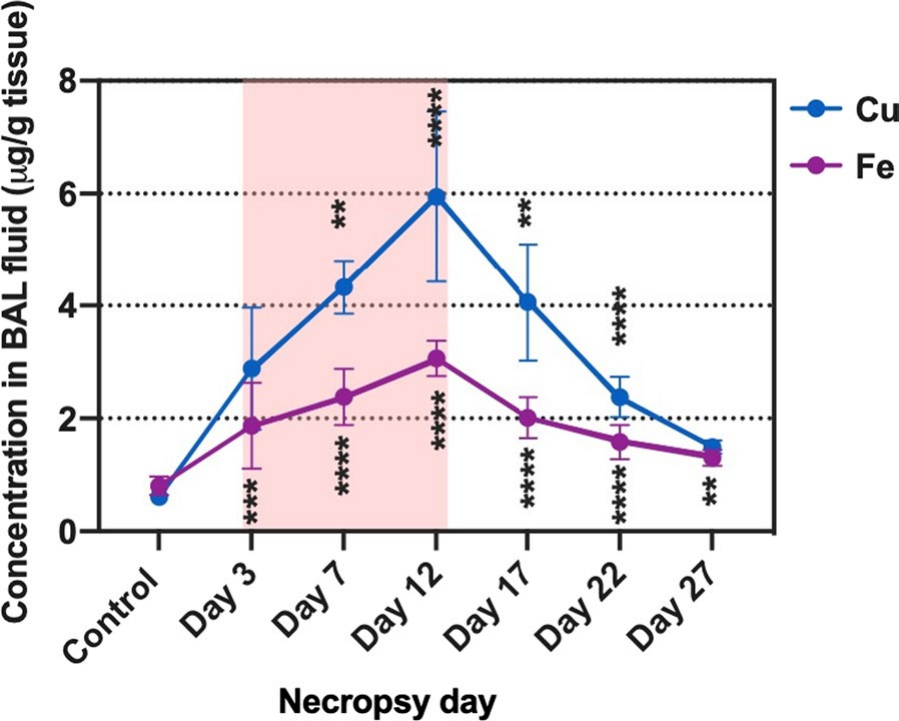
The concentration versus time profile of copper and iron in BAL fluid during (red-highlighted area) and after exposure. Statistical analysis of results for Fe in BAL fluid was performed using one-way ANOVA with Dunnett’s post hoc test, while statistical analysis of results for Cu in BAL fluid was performed by Kruskal–Wallis test (compare to the control group). Data are expressed as mean ± SD (n = 5). ****P < 0.0001, *** P < 0.001, **P < 0.01

Cu concentrations in lung tissue significantly increased at all-time points compared to the control group. Cu concentrations were elevated during exposure and started declining after exposure, which indicate a translocation and elimination of Cu out of the lungs. A log-Cu concentration versus time profile indicated first-order kinetics after day 12, and the elimination half-life (T_1/2_) at the exposure site was 6.5 days (Fig. 8). Total measured lung burden of Cu in the lung per mouse at day 12 (measured using ICP-MS) which was the last day of exposure was 10.1 ± 1.0 μg/mouse lung, while the estimated nominal lung burden of Cu was 53 μg/mouse lung. There were no significant changes in concentration of other elements in the lung tissues. Cu and Fe concentrations in BAL fluid increased during exposure and subsequently declined after exposure (Fig. 9). The percentage of soluble Cu in BAL fluid to the total Cu in lung tissue was less than 2.6% (Additional file 2: Table S1).

**Fig. 10:**
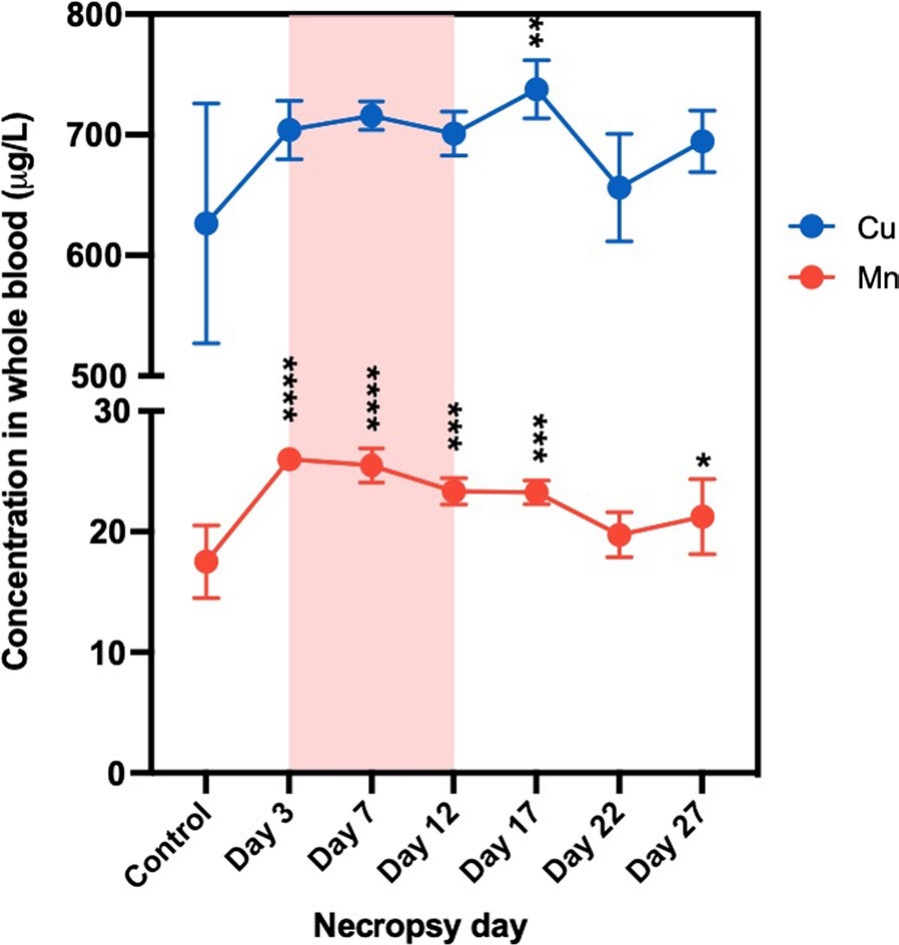
The concentration versus time profile in whole blood of Cu and Mn during (red-highlighted area) and after exposure. Statistical analysis of Mn was performed using one-way ANOVA with Dunnett’s post hoc test, while that of Cu was performed by Kruskal–Wallis test (compare to the control group). Data are expressed as mean ± SD (n = 5). ****P < 0.0001, *** P < 0.001, **P < 0.01, *P < 0.05

Whole blood samples showed significant concentration changes in 2 elements: Cu and Mn (Fig. 10). Cu concentration in blood showed an increasing trend during and after exposure with significant increases on day 17 (p < 0.01). This increase indicated that Cu ions could be absorbed into the bloodstream. Interestingly, Mn levels were substantially elevated at all-time points except on day 22 (Fig. 10).

**Fig. 11:**
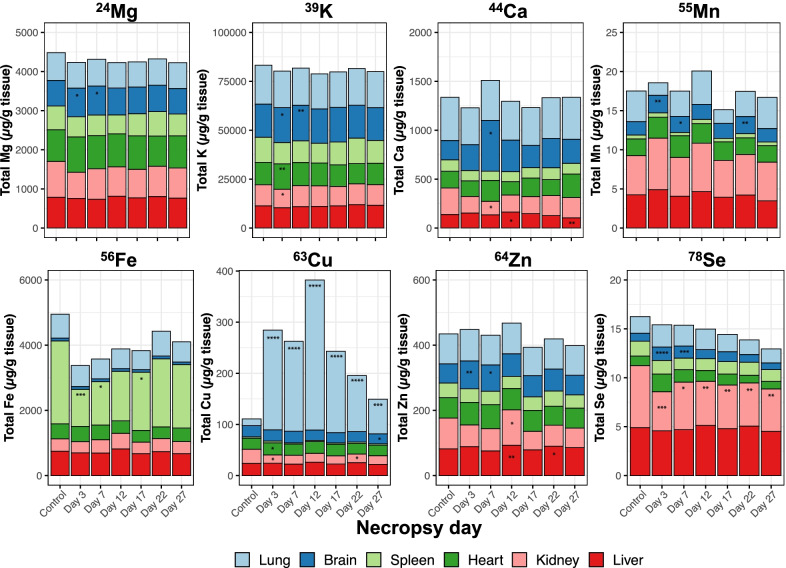
The concentration of each element in brain, heart, spleen, liver, kidneys, and lung; **a** Mg, **b** K, **c** Ca, **d** Mn, **e** Fe, **f** Cu, **g** Zn, and **h** Se. Statistical analysis was performed using one-way ANOVA with Dunnett’s post hoc test if the data were normally distributed. If not, Kruskal–Wallis test was used (compared to the control group). Data are expressed as mean ± SD (n = 5). ****P < 0.0001, *** P < 0.001, **P < 0.01, *P < 0.05

**Fig. 12:**
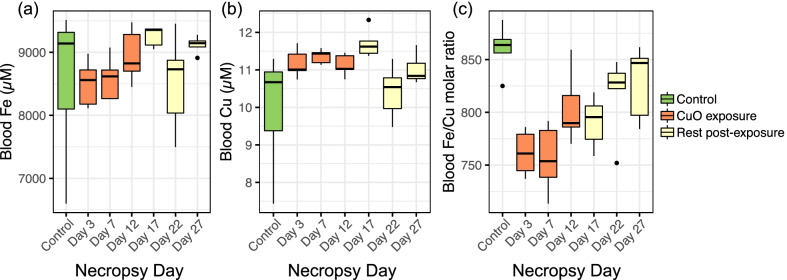
Blood concentration of **a** Fe, **b** Cu and **c** ratio of Fe/Cu. Black dots indicate outliers

**Fig. 13:**
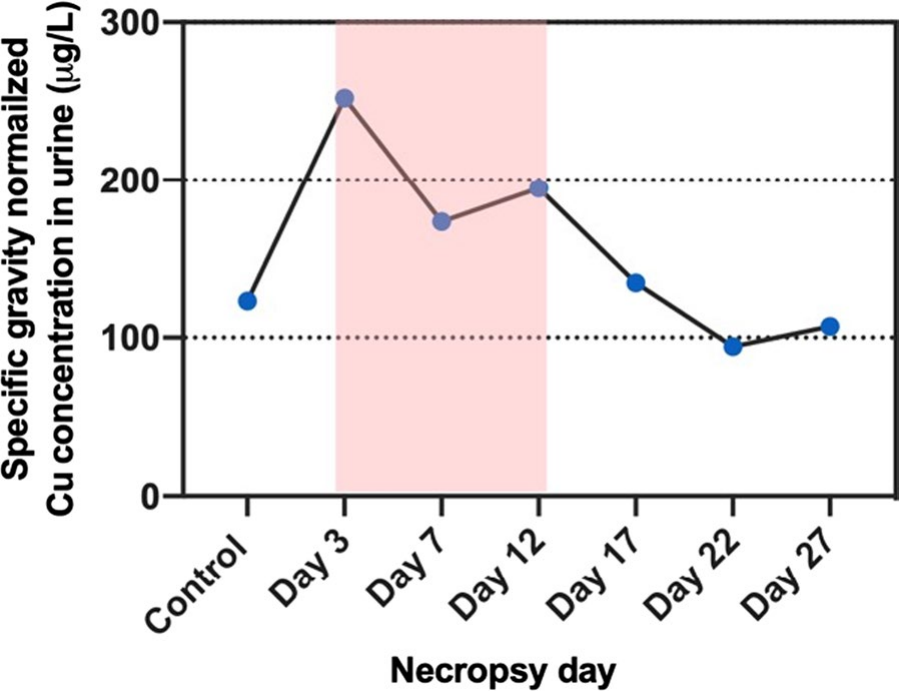
Cu concentration (µg/mL) in pooled urine samples during (red-highlighted area) and after exposure

The concentration of each element in the brain, heart, spleen, liver, kidneys, and lung was shown in stack plots (Fig. 11; (a) Mg, (b) K, (c) Ca, (d) Mn, (e) Fe, (f) Cu, (g) Zn, and (h) Se). Mean body weights and dried organ weights are listed in Additional file 3: Table S2. The concentration of Mg, K, Ca, Mn, and Zn were mostly stable over the experimental time. Fe concentration in the spleen was significantly decreased on day 3 (p < 0.001), day 7 (p < 0.05), and day 17 (p < 0.05) (Fig. 11e). Fe depletion in the spleen led us to evaluate the Fe levels in the blood. We found a slightly decreasing trend in Fe concentrations while an increasing trend in Cu concentrations in whole blood (Fig. 12a, b). This finding could indicate an interaction between Fe and Cu. The Fe to Cu molar ratio in whole blood decreased during exposure and returned to normal levels (i.e. as found in unexposed mice) (Fig. 12c). Analyses of Cu concentrations in the liver and kidneys showed no significant increases compared to controls. Levels of Cu ions in kidneys significantly decreased compared to controls on days 3 and 17 (Fig. 11f). Cu concentration increased in heart tissue during exposure with statistical significance only on Day 3 (p < 0.05) (Fig. 11f). Significant decreases in Se concentrations were found at all time points in kidneys, which indicates that inhaled Cu led to a negative impact or might be excreted by the kidney (Fig. 11h). This excretion route was confirmed by analyzing Cu ions in pooled urine samples. The concentration of Cu in urine was normalized using specific gravity to reduce the variability in urine dilution [30]. The specific gravity showed higher values during CuO exposure than the resting period, which was possibly caused by water restriction during the 4-h-nose-only inhalation exposure (Additional file 4: Table S3). Cu concentrations in urine increased during exposure days and returned close to the concentration of the control group by day 17 (Fig. 13). Several elements showed significant changes in the brain tissue, such as Mg, K, Mn, Zn, and Se. These changes mainly occurred on day 3 and 7, when mice were being exposed to CuO NP aerosol (Fig. 11).

### Hemoglobin and ceruloplasmin levels in serum samples

**Fig. 14:**
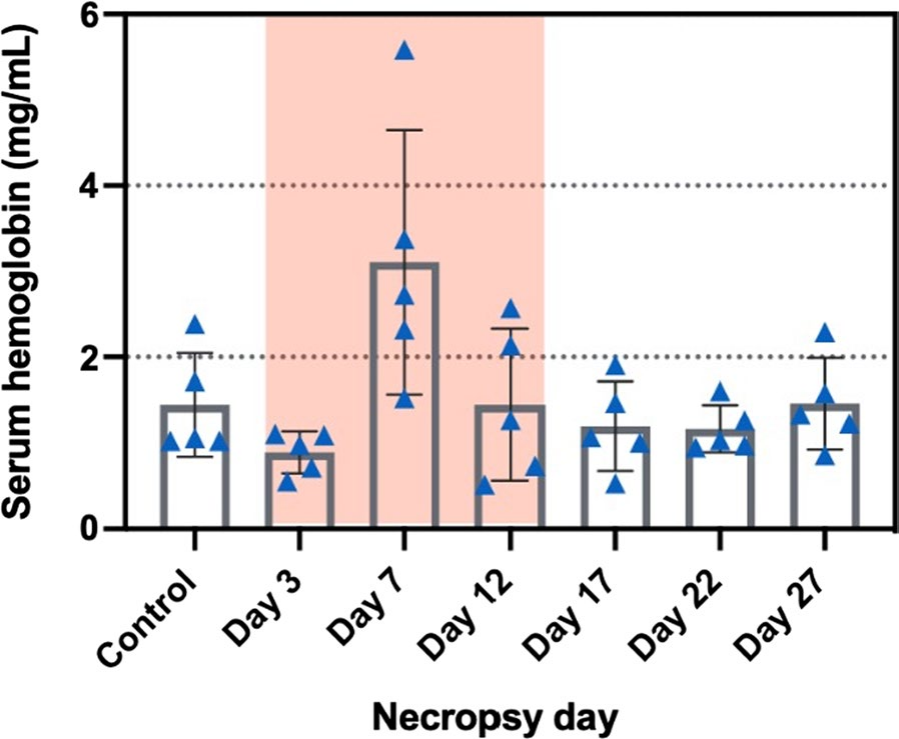
Serum hemoglobin levels (mg/mL) during (red-highlighted area) and after exposure. Statistical analysis was performed by Kruskal–Wallis test (compared to the control group). There was no significant difference between the control and exposed mice. Data are expressed as mean ± SD (n = 5)

**Fig. 15:**
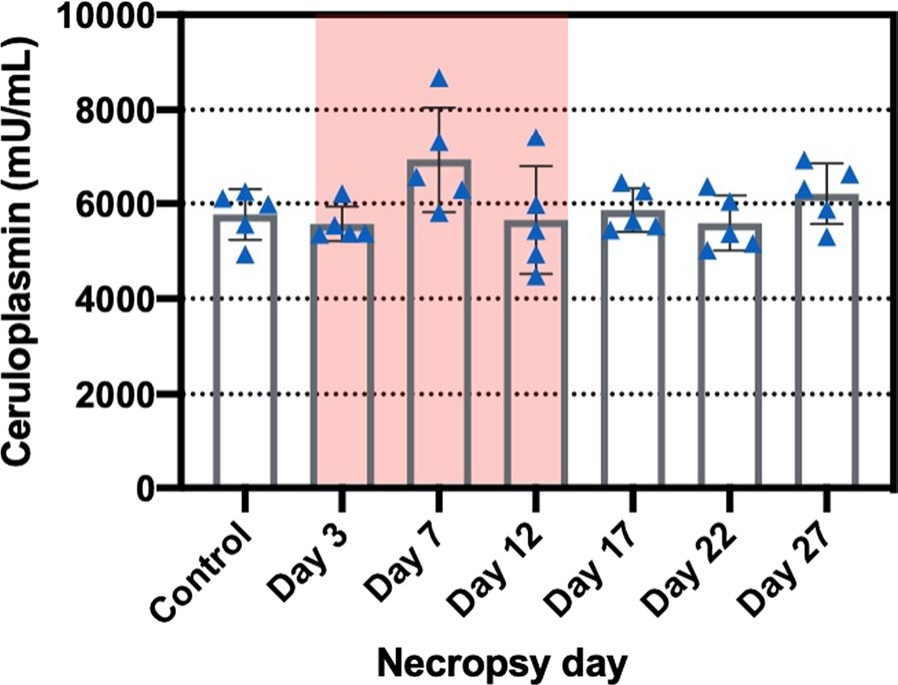
Serum ceruloplasmin level (mU/mL) during (red-highlighted area) and after exposure. Statistical analysis was performed using one-way ANOVA with Dunnett’s post hoc test. There was no significant difference between the control and exposed mice. Data are expressed as mean ± SD (n = 5)

Hemoglobin concentrations from serum samples showed an increase only on day 7, but without reaching significance (Fig. 14), which was not correlated in Fe/Cu ratio changes in whole blood. Serum ceruloplasmin concentrations showed a slight increase also on day 7, but this change was not significantly different from controls, due to high variability of measured values on this day (Fig. 15).

### Body weight and dry organ weight changes

**Fig. 16:**
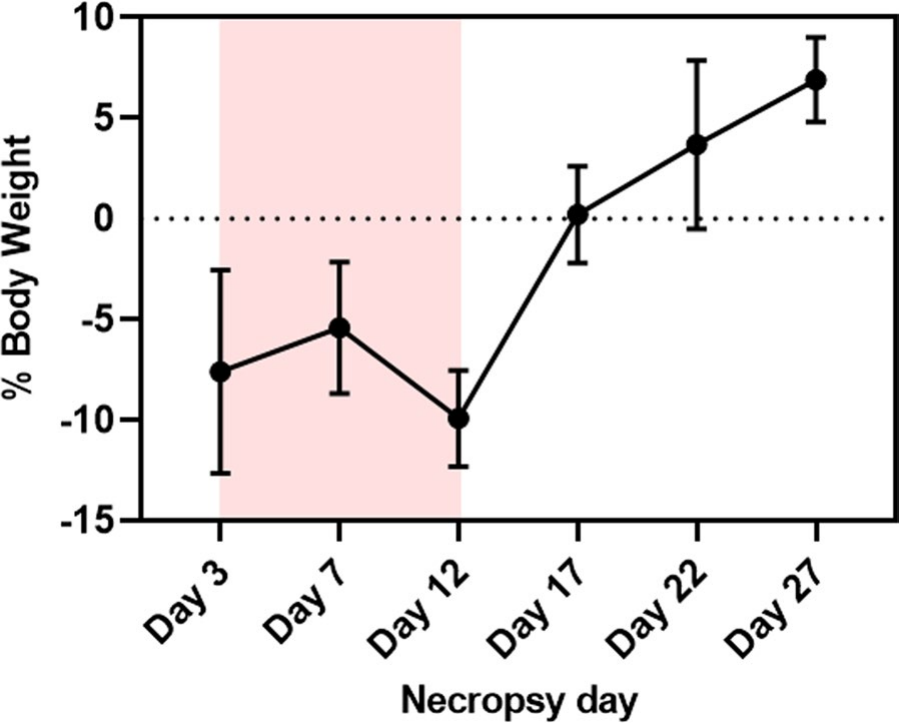
Body weight as a relative percentage of starting weight ("0%") during (red-highlighted area) and after exposure

**Fig. 17:**
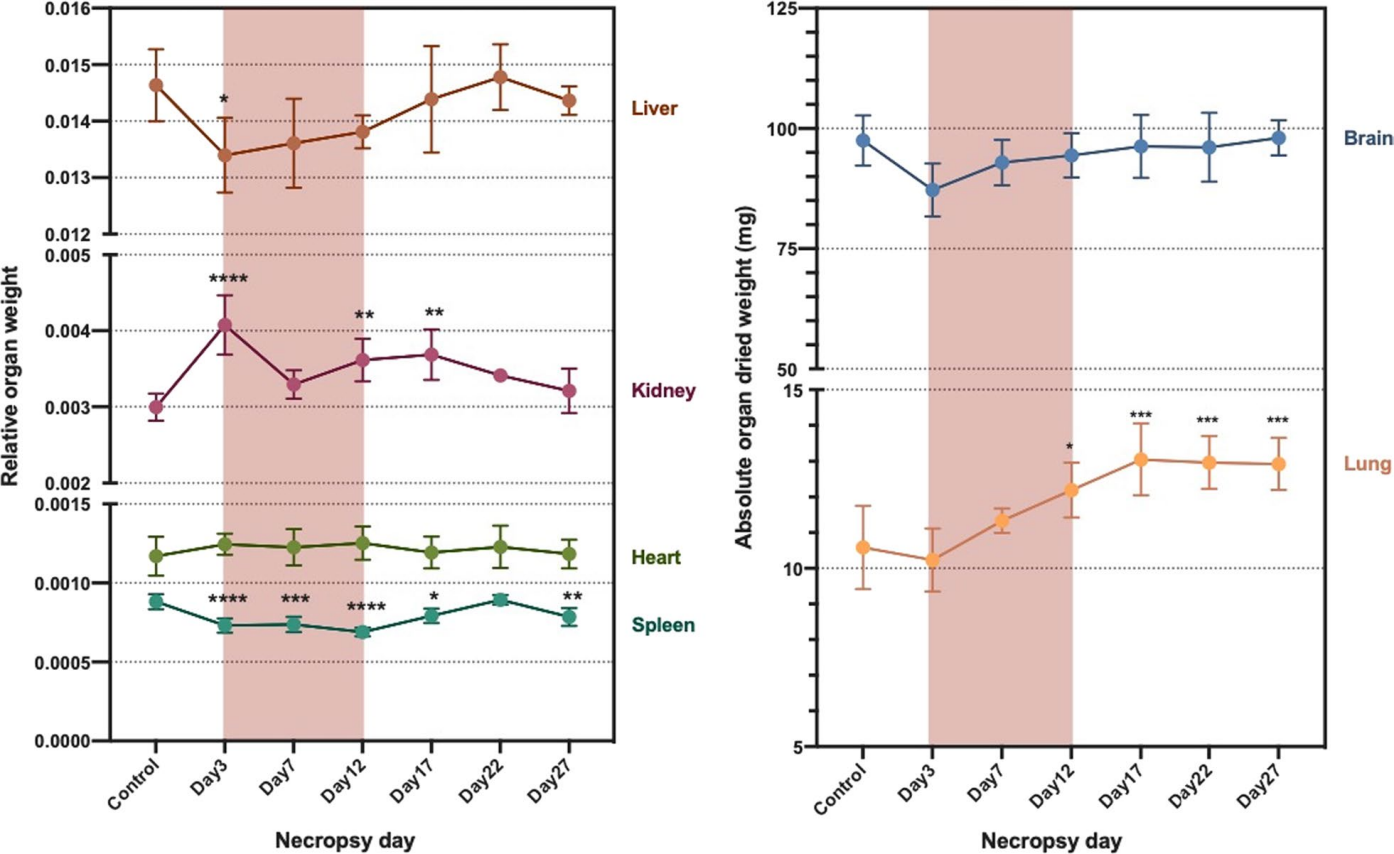
Organ dry weight change during (red-highlighted area) and after exposure. Statistical analysis of results for liver, kidney, heart, spleen, and lung was performed using one-way ANOVA with Dunnett’s post hoc test, while statistical analysis of results for brain was performed by Kruskal–Wallis test (compared to the control group). Data are expressed as mean ± SD (n = 5). ****P < 0.0001, *** P < 0.001, **P < 0.01, *P < 0.05

Body weight decreased at days 3, 7, and 12, which corresponded to the time when mice were exposed to CuO NP aerosols. After exposure was stopped, body weight increased at subsequent time points (Fig. 16). Significant changes in organ weights were observed for kidneys, spleen, and lung (Fig. 17). Relative kidney weight showed significant increases on day 3 (p < 0.0001), day 12 (p < 0.01), and day 17 (p < 0.01) where the weight increased approximately 3.2–10.9% compared to the control group. On the other hand, relative spleen weight showed significant decreases at all time points except day 22 (p < 0.0001 on day 3 and 12, p < 0.001 on day 7, p < 0.01 on day 27, and p < 0.05 on day 17). Spleen dry weight during exposure decreased by 31.7–38.6% compared to spleens from the control group. Absolute lung weight was significantly increased after exposure; approximately 15.2–23.3% compared to lungs from the control group (p < 0.001 on day 17, 22, and 27, p < 0.05 on day 12).

### Dissolution of CuO NPs in simulated biological fluids

Dissolution studies on CuO NPs were conducted in 3 artificial biological fluids including ALF, SELF, and SGF to determine their potential toxicity at specific sites since it has been shown that the toxicity of CuO NPs is mainly influenced by the release of Cu ions [31]. CuO NPs were completely dissolved in SGF within 4 h, and ALF within 14 days. In contrast, only up to 5% of CuO NPs in SELF fluid was dissolved after 14 days (Additional file 5: Figure S2).

## Discussion

This study focused on three aims: (1) to observe the time course of pulmonary inflammation during and after CuO NP sub-acute inhalation exposure, (2) to determine the biodistribution of Cu and its clearance rate from the lungs, and (3) to investigate the effect of inhaled Cu on secondary organs by monitoring concentrations of trace elements and organ weights.

There were three major findings from these studies. First, sub-acute inhalation of CuO NPs (an estimated lung burden of 53 µg/mouse not considering lung clearance) led to pulmonary cytotoxicity (elevated LDH concentrations in BAL fluid) and inflammation (increased total cell numbers, neutrophils, and macrophages, and inflammatory cytokines in BAL fluid and perivascular chronic inflammation in lung tissue). Observed progressive inflammation corresponded to the Cu concentration in lung tissue, and soluble Cu in BAL fluid, which started increasing during exposure and subsequently decreased post-exposure. The airway inflammation was still present at least 15 days post-exposure (day 27). Second, as expected, the lung as the route given the study design was the main organ that showed significant increases in Cu concentrations compared to controls at every time point. Cu concentrations declined in lungs with first-order kinetics and an elimination T_1/2_ of 6.5 days. Finally, disruption in trace element homeostasis was observed in three organs: spleen, kidneys, and brain. Significant organ weight changes were found in kidneys, spleen, and lung.

Inflammatory responses in lung after inhalation of CuO NPs have been recognized by many studies of ours [19, 32, 33] and others [17]. The experimental design in most of these studies investigated fewer time points and only after exposure. To the best of our knowledge, the effect of inhaled CuO NPs over time on pulmonary inflammation, biodistribution, and impact on trace elements in secondary organs has not been investigated, even though inflammation may disrupt the homeostasis of trace elements in the organism (whole blood or tissues) [34]. In this study, mice were exposed to an estimated dose of 53 μg/mouse (2.65 mg/kg) which is equivalent to 168 mg/human (assuming mouse body weight of 20 g and human body weight of 70 kg). The dose of 168 mg/human is equivalent to 16 work weeks of exposure assuming the exposure concentration of 1 mg/m^3^ (current OSHA PEL and NIOSH REL for copper dust and mist (excluding fumes)), breathing frequency of 15 breaths/min, tidal volume of 600 mL/breath, and pulmonary deposition fraction for 36 nm particles of 0.5.

Neutrophils in BAL fluid significantly increased at all time points but maximally increased during exposure and declined after exposure. Similarly, as in our previous studies, neutrophils were the major cells recruited to the lung after inhalation exposure to Cu or CuO NPs [13, 19, 32] Alveolar macrophages decreased during exposure and subsequently increased after exposure. The decrease in macrophage numbers during exposure could be due to particulate clearance mechanisms by phagocytosis and mucociliary escalator. Another possibility is due to highly dissolved Cu in lysosomal fluid inducing cell damage as our dissolution study showed that CuO NPs dissolved rapidly in ALF. Areecheewakul et al. showed that mice exposed to CuO NP aerosols at 3.5 mg/m^3^ for 4 h exhibited decreases in macrophages viability [13], in this study we only have data on the viability of total cells which did not decrease over time in comparison to controls (data not shown). Gosens et al. investigated the organ burden and pulmonary toxicity of inhaled CuO NP concentrations for a short-term (5 days) exposure period in rats and their findings supported our observations with respect to the elevation of LDH concentrations, total cell counts, and the number of neutrophils, macrophages, and lymphocytes [17]. Increases in concentrations of cytokines/chemokines such as KC, G-CSF, GM-CSF, MCP-1, MIP-1α, and MIP-1β went along with the observed increases in the number of neutrophils and macrophages. Moreover, cytokines related to damaged lung epithelial cells, IL-1α and IL-6, elevated during exposure, corresponding to the high degree of inflammation during exposure. The results from inflammatory cells and cytokines indicated that inhaled Cu predominantly affected innate immunity (changes in neutrophils, macrophages, and cytokines associated with innate immune cells). Holan et al. [35] performed a kinetic study of continuous three-month inhaled CuO NP exposure in mice at different time intervals (3, 14, 42, and 93 days) to evaluate the effect of inhaled CuO NPs on innate and adaptive immune responses. They found that inhalation of CuO NPs mainly influenced innate immune cells (changes in the percentages of eosinophils, neutrophils, macrophages, and antigen-presenting cells) with a minimal effect on the percentage of T and B lymphocytes; however, the effect on proliferation and secretory activity of T cells strongly depended on the duration of inhalation exposure [35]. We previously reported that inhalation exposure to CuO NP impaired murine host defense against instilled *Klebsiella pneumoniae* and induced a dose-dependent decrease in bacterial clearance from the lung [32]. In the present study, lung inflammation induced by CuO NP sub-acute inhalation exposure was still observed at 15 days post exposure as the number of total cells, neutrophils, and concentration of LDH in BAL fluid were still higher than the control, and perivascular inflammation persisted until this time.

The measured amount of Cu in the lung at day 12 was fivefold lower than the estimated nominal lung burden of 53 μg/mouse. This discrepancy may be attributed to this measure excluding lung clearance, or translocation to extrapulmonary organs, in the estimation of deposition fraction of Cu from MPPD modeling. The measured soluble Cu in BAL fluid was less than 2.6% of total Cu in lung, indicating CuO NPs were slightly dissolved in BAL fluid (Additional file 2: Table S1). This finding corresponded to our dissolution studies where we found that dissolution of CuO NPs in SELF was very low (~ 5% after 14 days). These results further indicate that CuO NPs were taken up by the lung tissue in the form of NPs. A fraction of inhaled particles deposited in the alveolar region can be removed by mucociliary clearance and enter the gastrointestinal tract (GIT). Our dissolution studies in SGF (Additional file 5: Figure S2) with very low pH (1.5) revealed that CuO NPs would dissolve completely and facilitate absorption of ionic Cu into the blood by active or passive mechanisms. Fate and bioavailability of CuO NPs from GIT may cause changes in the composition and function of gut microbiota, in trace element levels in extrapulmonary organs or whole blood, or cause systemic changes in the organism [34, 36, 37]. Further investigation of adverse effects of NPs on GIT and extrapulmonary organs after inhalation exposure is warranted.

Iron is essential to many neutrophil inflammatory responses [34]. Increases in Fe concentrations in the BAL fluid at all time points could be indicative of lung injury. Numerous clinical studies showed strong correlations between Fe levels in BAL fluid and the presence or severity of acute respiratory distress syndrome (ARDS) [38], which is an acute inflammation with neutrophil infiltration, increased vascular permeability, and diffuse alveolar damage [39]. The concentration of Fe in our study correlated well with the increased number of neutrophils. Acute lung injury can lead to a compromised pulmonary vascular compartment where RBC could escape and then lyse in the airways [40], however ARDS was not observed based on histopathology evaluation.

Whole blood analysis showed an increase in Cu concentration during exposure, indicating that inhaled Cu may undergo a dissolution and could be absorbed into the bloodstream. This finding is potentially supported by an increasing trend in Cu concentrations in heart during the exposure, even though this increase was statistically significant only on day 3.

According to analysis of other trace elements in secondary organs, Fe depletion in the spleen was clearly observed during exposure demonstrating that inhaled Cu perturbs Fe homeostasis in the spleen. The Fe to Cu molar ratio in whole blood decreased during exposure and then returned to normal levels (as seen in unexposed mice), which led us to investigate hemolysis induced by Cu. Several studies reported that Cu could induce hemolysis [41–44]. Copper chloride at a concentration of 30 µM can induce 50% hemolysis of mouse erythrocytes in phosphate buffer saline after 2.5 h at 37 °C [41]. Wilson’s disease, an inherited disorder of Cu metabolism, exhibits excessive Cu in the blood circulation resulting in hemolytic anemia [42, 43]. In our study, we only found an increase in hemoglobin levels on day 7 (without significance). Mn concentrations in whole blood demonstrated an apparent increase during exposure. Studies conducted by Mercadante et al. showed that rats exposed orally to 11.1 mg Mn/kg body weight for 7 or 61 days could perturb Fe, Cu, or Zn tissue concentrations but the most prominent effect was on Cu [45]. An occupational study of Mn-exposed smelter workers showed Cu concentrations in whole blood are significantly higher in exposed groups than in controls [46]. These studies suggested Mn exposure can affect Cu homeostasis. The impact of the increase of one metal element on others might be because trace metal elements often share similar transport mechanisms [47]. For example, divalent metal transporter-1 (DMT) functions as a major Fe transporter and transporter for other heavy metal ions, including Mn^2+^, Cu^2+^, and Co^2+^ [48]. Selenium concentrations in the kidney significantly decreased at all time points, which could be a sign of kidney injury even though we did not detect significant Cu concentration changes in this organ. Kidneys contain the highest Se concentrations among all organs and renal injury often occurs together with hyposelenemia [49, 50]. Lai et al. demonstrated that Se deficiency elevated oxidative stress resulting in mitochondria damage and led to renal injury in mice [50]. Pooled urine samples were also analyzed, and it was found that there were increased Cu concentrations during exposure. According to the dosimetry data, exposure to inhaled CuO NP caused elevations of Cu in the lung, blood stream, and heart. Liu et al. investigated Cu NPs biodistribution in mice after nasal instillation (exposure at 40 mg/kg body weight for 3 times in 1 week) and showed that Cu NPs can translocate to liver, kidneys, and the olfactory bulb [51]. Our inductively coupled plasma-mass spectrometer (ICP-MS) analysis in brain tissue did not show changes in Cu concentrations, however, levels of several other elements such as Mg, K, Ca, Mn, Zn, and Se were significantly altered. This indicates that inhalation of CuO NPs can disrupt trace element homeostasis in brain. Bai et al [52] supported our findings as they showed that intranasally instilled Cu NPs modified levels of Zn, Ca, and Fe in brain. Mn in brain in our study was significantly elevated compared to controls. Mn induces oxidative stress and neuroinflammation and dysregulates multiple neurotransmitters associated with motor deficits, learning, memory and cognition [53] and thus inhalation of CuO NPs may cause neurotoxicity indirectly through disruption of trace element transporter which should be investigated further [54].

Ceruloplasmin, an α-2-glycoprotein containing 95% of the Cu in plasma, functions as a major Cu transporter protein and multi-Cu oxidase protein, it mobilizes Fe from Fe storage cells into plasma [55]. It is an acute-phase plasma protein produced by hepatocytes and activated monocytes and macrophages and serves as an antioxidant that is often increased in inflammatory conditions [56–58]. Several studies have found a positive correlation between serum Cu and ceruloplasmin [59–62]. It has been suggested that the serum ceruloplasmin levels can serve as a biomarker for copper exposure. Serum Cu and ceruloplasmin levels from employees of a copper handling industry were found to have a statistically significant positive correlation in cases of chronic moderate occupational exposure to Cu [62]. Our study did not find this positive correlation. This could be because our Cu exposure did not induce sufficient increase in serum Cu levels to produce an increase in ceruloplasmin. In addition, our exposure time might not have been long enough to cause significant change in ceruloplasmin levels and/or it is possible that homeostatic mechanisms still functioned adequately to prevent significant changes [63]. In our study, the concentration of ceruloplasmin in serum was highest on exposure day 7, however this increase was not significant compared to controls. Seeing these results, we would expect that the levels of ceruloplasmin would stay elevated also on Day 12, if it would be correlated with pulmonary inflammation, but this was not the case. It appears that CuO NP inhalation exposure caused inflammatory effects that are more localized in the lungs rather than systemic effects as seen in our previous study where we found much lower concentrations of pro-inflammatory cytokines in plasma of CuO-NP-exposed mice compared to levels in BAL fluid [33].

Relative organ weight has been accepted as a valuable biomarker in toxicological studies to identify the potentially harmful effect of exposed substances [64, 65]. Absolute lung weights of exposed mice were higher than that of control mice at all time points, particularly at the post exposure phase. This supports our finding that lung inflammation developed soon after the exposure was commenced and continued through to 15 days post exposure. Wahlström et al. demonstrated that increased lung weight correlated to the histopathological findings and the degree of inflammatory lesions, alveolar macrophages, or perivascular inflammation [66]. Relative spleen and kidney weights of exposed mice respectively decreased and increased significantly during exposure which indicated that inhaled Cu could have negative effects on these organs.

Dissolution studies of CuO NPs conducted to help understand and predict toxicity showed that CuO NPs were dissolved in ALF and SGF, but dissolution in SELF was very low. Similar to Pettibone et al. and Lee et al., Cu NPs used in this study dissolved in ALF, SELF, and SGF at 100%, 2%, and 84% within 24 h, respectively [19, 31]. Fast dissolution (~ 80% within 24 h) in conditions simulating the acidic milieu of phagolysosomes (ALF) indicated that Cu NPs will not persist inside the macrophages in the particulate form for a very long time. This was supported by the appearance of particulates inside the macrophages in BAL fluid (Fig. 5). Particulate agglomerates/aggregates were visible at earlier time points at day 3–12, but not at later time points when daily exposures stopped.

## Conclusions

Sub-acute inhalation exposure to CuO NPs in BALB/c mice led to pulmonary toxicity represented by elevations of LDH, total cell number, neutrophils, macrophages, and inflammatory cytokines. The degree of pulmonary inflammation corresponded to the total amount of Cu in lung tissue, soluble Cu in BAL fluid, and Fe in BAL fluid, indicating that lung injury further increased during exposure and gradually declined after exposure. Decline in Cu concentrations in the lung post exposure exhibited as first-order kinetics. Whole blood and heart tissue showed marginal but significant increases in total Cu ion concentrations during exposure, exhibiting that dissolved Cu ions were absorbed into the bloodstream and heart tissue. The acute pulmonary toxicity was also observed by significant increases in the lung dry weight of exposed mice. Secondary organs affected by CuO NPs subacute inhalation were kidneys and spleen as they showed changes in organ weight and trace elements.

This study confirmed our previous findings of a robust but reversible pulmonary inflammatory potential of CuO NPs after inhalation, which was correlated to Cu concentrations in the lungs. The findings on disruption of trace element homeostasis and organ weight changes in the kidneys and spleen suggest further investigation into additional toxicity outcomes on these organs caused by CuO NP inhalation exposure is warranted.

## Methods

### CuO NP dispersion and animal inhalation exposure

Female BALB/c mice (4-week-old) from Jackson Laboratories (Bar Harbor, ME) were acclimatized in the vivarium with a 12-h light/dark cycle with ad libitum access to food and water for 7 days before inhalation exposure. Animal protocols performed were approved by the University of Iowa Institutional Animal Care and Use Committee. This mouse strain was selected to complement other studies from our laboratory focused on a deeper investigation of an immunomodulatory effects of CuO NPs in asthmatic and allergen immunotherapy mouse models in females. In this work we sought to determine phases of pulmonary inflammation initiation, progression, and resolution.

Exposures to CuO NP aerosol were performed using a nose-only inExpose system (SCIREQ Inc., Emka Technologies, Montreal, Canada). To minimize the stress induced by the exposure system, mice were acclimated to the nose-only holders for 3 days before the start of the exposure by placing them into nose-only holders connected to a tower with filtered air flow for an increasing time on each day as follows: 30 min, 1 h, and 3 h per day, respectively. Subsequently mice were exposed to CuO NP aerosols for 4 h/day from day 1 to day 12 with 2 days interruption on days 6 and 7. CuO NPs were provided and characterized by Nanomaterials Health Implications Research (NHIR) at the Engineered Nanomaterials Resource and Coordination Core (ERCC), HSPH-NIEHS Nanosafety Center, Harvard T.H. Chan School of Public Health, member of the Nanomaterials Health Implications Research (NHIR) Consortium. Nanoaerosols were generated as described previously [13]. Dry powder consisting of CuO NPs was suspended in distilled water at 1 mg/mL and sonicated using a cup horn sonicator (QSonica, CT) at 75% amplitude for 5 min. The dissolution of CuO NPs in water after sonication was found to be minimal. There was only 0.0001% of Cu ions detected by ICP-MS from total CuO NPs suspended in water immediately after sonication. The dissolution of CuO NPs was monitored every 0.5 h up to 4 h, there was 0.001% dissolved Cu from total suspended NPs within 1 h and 0.002% within 2 h, and it stayed at this level at the 4 h time point. The suspension was transferred to a glass nebulizer jar with a magnetic bar and placed on a magnetic stirrer to prevent CuO NP sedimentation during inhalation exposure. CuO NP aerosols were generated using a 6-jet Collison nebulizer (BGI Inc., Waltham, MA) supplied with dehumidified and HEPA-filtered air. The generated aerosol was passed through a brass drying column heated at 110 °C, a humidity condensation jar, and a particle neutralizer (containing 10 mCi ^85^Kr source, TSI Inc., Shoreview, MN) prior to splitting and entering two nose-only inExpose towers each holding 12 mice.

The CuO NP aerosol concentration was monitored during the exposure using a direct-reading aerosol photometer (DataRAM™ pDR-1500, Thermo Fisher Scientific Inc., Waltham, MA) to aid in controlling the concentration near the target concentration of 3.5 mg/m^3^. The selection of exposure concentration was based on our previous studies [32, 33, 67], as well as considering relevance to potential human exposures. The pDR was calibrated for CuO NP aerosol prior the start of the study. Time-weighted average (TWA) concentration was determined gravimetrically using pre-weighed 37-mm glass microfiber filters (Whatman 1827–037; Middlesex, UK) that were incorporated into the pDR and a six-place microbalance (Mettler Toledo XP26; Columbus, OH) in a climate-controlled laboratory. The particle size distribution of the generated aerosol was measured using a SMPS (TSI Inc., Shoreview, MN) and presented as GM and GSD (Fig. 1).

To study sub-acute inhalation effects, mice were exposed for 4 h/day, 5 days/week for 2 weeks. Sentinel mice were used as controls for all comparisons. Mice were euthanized with an overdose of isoflurane, followed by cervical dislocation, thoracotomy, and exsanguination through the heart on day 0, 3, 7, 12, 17, 22, and 27 (n = 5 per time point). Controls were necropsied 3 days after the last time point investigated. Day 3, 7, and 12 were the days of CuO NP exposure period. BAL fluid was collected from the right lobes of the lung to assess the occurrence of pulmonary inflammation induced by CuO NP aerosols. The right lobes of the lung were perfused with 10% buffered formalin (Fisher Scientific, Kalamazoo, MI) through the cannulated trachea and then stored in 10% buffered formalin until further processing for histopathology. Dosimetry analysis for trace elements was performed on whole blood, left lobes of lung, brain, heart, spleen, liver, kidneys, BAL fluid, and urine. Whole blood was kept in blood collection tubes containing ethylenediaminetetraacetic acid (EDTA) as a chelating agent and stored at -80 °C. All organs were frozen in liquid nitrogen immediately after collection and stored at -80 °C. Urine samples were collected directly from the bladder with a syringe at necropsy. Since not all mice had urine in the bladder and due to limited volume of urine from each mouse, urine samples from mice in the same group were pooled for dosimetry analysis using ICP-MS.

### Estimated dose

The estimated deposited dose in the pulmonary region was calculated using the equation below:

$$D_{m} = C \times RMV \times t \times \alpha$$

where D_m_, Dose per mouse (× 10^–3^ µg/mouse), C: Concentration (mg/m^3^), RMV, Respiratory minute volume (mL/min), t, Exposure time (min), $\alpha$, Pulmonary deposition fraction.

The average concentration of CuO NP aerosol over all exposure days was 3.75 mg/m^3^. Considering breathing frequency of 165 breaths/min and tidal volume of 0.15 mL, the respiratory minute volume considered for this calculation was 24.8 mL/min. The pulmonary deposition fraction not considering clearance was calculated using the computational multiple path particle dosimetry (MPPD) model (software version 3.04 (Applied research associates (ARA), Albuquerque, NM) which was 0.24 [68]. Thus, estimated nominal dose of CuO NPs deposited in the pulmonary region was 53 µg/mouse.

### Assessment of pulmonary inflammation from bronchoalveolar lavage (BAL) fluid

BAL fluid was collected from the right lobes of the lung by lavaging with 1 mL sterile sodium chloride solution (0.9%, Baxter, Deerfield, IL) 3 times. The collected BAL fluid was centrifuged at 800 × *g* for 5 min at 4 °C to separate the cellular and soluble components. The supernatant was stored at -80 °C to analyze LDH enzyme levels and cytokine/chemokine concentrations. Cell pellets from BAL fluid were resuspended in 200 µL Hank’s balanced salt solution (Life Technologies, Grand Island, NY) and stained with propidium iodide (PI) and the number of viable and dead cells counted using an automated cell counter (Moxi GO II, Orflo Technologies, LLC, Ketchum, ID). Briefly, 135 µL of 2.25 µg/mL PI in phosphate buffered saline, pH 7.4 containing 0.045% sodium azide (Moxi Cyte Viability Reagent, Orflo technologies, Ketchum, ID) was added to 15 µL cell suspension, and incubated for 5 min. Then, 75 µL of sample was loaded into the MFS cassette (MXC020). The total viable cell count was measured using a fluorescence laser at 488 nm with a 561/LP filter. For differential cell counts, cells were fixed on microscope slides with fetal calf serum using a Cytospin™ 4 Cytocentrifuge (Thermo Shandon, Thermo Scientific, Waltham, MA), and stained using the Protocol® HEMA 3 stain set (Fisher Diagnostics, Pittsburgh, PA), which is similar to the traditional Wright and Wright-Giemsa stains. Cells were differentiated by type (macrophages, lymphocytes, neutrophils, and eosinophils) in 400 cell counts per sample using a bright-field mode microscope (Olympus, Center Valley, PA).

The levels of soluble substances in BAL fluid such as LDH and inflammatory cytokines/ chemokines were determined. LDH, a cytosolic enzyme released from cells upon cell membrane damage was measured as an indicator of cytotoxicity within the pulmonary region. LDH was spectrophotometrically measured using a cytotoxicity detection kit LDH (Roche Diagnostics, Indianapolis, IN). The following cytokines in the supernatant of BAL fluid; eotaxin, G-CSF, GM-CSF, IFN-γ, IL-1α, IL-1β, IL-2, IL-3, IL-4, IL-5, IL-6, IL-9, IL-10, IL-12(p40), IL-12(p70), IL-13, IL-17A, KC, MCP-1, MIP-1α, MIP-1β, RANTES, and TNF-α were simultaneously determined using Bio-Plex Pro™ mouse cytokine 23-plex assay (Bio-Rad laboratories, Hercules, CA) according to the manufacturer’s protocol and measured using the Bio-Plex 200 system (Bio-Rad).

### Lung histopathology

After cardiac exsanguination and BAL fluid collection, the right lobes of the lung were perfused with 10% buffered formalin (Fisher Scientific, Kalamazoo, MI) through the cannulated trachea and then stored in 10% buffered formalin until further processing. The methods for lung histopathology have been previously described elsewhere [13, 69].

### Trace element analysis in whole blood, organs, and urine

The following elements were quantified in whole blood, lung, brain, heart, spleen, liver, kidneys, BAL fluid, and urine: Cu (^63^Cu), potassium (^39^K), magnesium (^24^Mg), calcium (^44^Ca), manganese (^55^Mn), Fe (^56^Fe), zinc (^64^Zn), and selenium (^78^Se) using ICP-MS (Agilent 7900, Santa Clara, CA) with helium gas in collision cell mode. Yttrium (^70^Y), rhodium (^103^Rh), tellurium (^130^Te), iridium (^193^Ir), and bismuth (^209^Bi) were utilized as internal standards. All reagents used for ICP-MS measurement are listed in Additional file 6: Table S4.

### Whole blood analysis

The Centers for Disease Control and Prevention (CDC) method developed by Jones et al., 2017 was applied to determine trace element concentration in whole blood after CuO NP inhalation exposure [70]. The diluent reagent consisted of 0.4% v/v tetramethylammonium hydroxide (TMAH), 1% v/v ethanol, 0.01% w/v ammonium pyrrolidine dithiocarbamate (APDC), 0.05% v/v triton X-100, and 5 μg/L of each Ir, Rh, Te (internal standard) in deionized (DI) water. Whole blood was vortexed for 15 s and diluted 50X with DI water and the diluent.

### Microwave-assisted acid digestion of tissues

All frozen organs were lyophilized using a freeze dryer (FreeZone, Freeze Dry Systems, Labconco Co., Kansas City, MO) for 24 h, and weighed using an analytical balance (Mettler Toledo, Mo. XPR206CDR). All tissue samples were digested using modified EPA method 3051 [71, 72] using trace metal grade reagents. Briefly, lyophilized organs were transferred to a pre-weighed Teflon digestion vessel consisting of 9 mL concentrated nitric acid (HNO_3_) and 1 mL 30% hydrogen peroxide (H_2_O_2_). The samples were acid digested using a microwave digestion system (Milestone ETHOS UP, Sorisole, Italy) set with a ramp to 210 °C for 20 min, and hold at 210 °C for 15 min. After microwave digestion, the samples were diluted with 40 mL deionized water. The digested samples were weighed with the digestion vessel and recorded as post-weighed samples to calculate the final solution mass. Acid-digested samples of the lung, kidney, and liver samples were diluted with 2% HNO_3_ 10 times, while that of the brain samples were diluted 5 times. Heart and spleen acid-digested samples were measured without further dilutions. Measured elemental concentrations were expressed as µg/g dry tissue weight.

BAL fluid collected from the right lobes was centrifuged at 194,400 × *g* (Beckman Coulter, Optima™ XPN-100) for 10 min to eliminate CuO NPs. The supernatants were diluted 5X with 2% HNO_3_ before ICP-MS measurement. The reported concentration of Cu represented soluble Cu in BAL fluid.

### Urine analysis

Cu and other trace elements in urine samples were measured using a method from the CDC’s Division of Laboratory Sciences (Method No. 3031.1–01). The diluent reagent consisted of 10 µg/L Rh as an internal standard in 2% v/v HNO_3_ and 1.5% v/v ethanol. Urine samples were diluted 20X with DI water and the diluent.

### Quality assurance and quality control of trace elements analysis

Our laboratory is enrolled in the Lead and Multielement Proficiency Program (LAMP), a laboratory standardization program run by the CDC [73]. Each quarter, our laboratory blindly analyzes a provided set of bovine blood samples and reports the results. In addition, blood and urine samples certified for additional elements under the Quebec Multielement External Quality Assessment Scheme (QMEQAS) were purchased from The Centre de Toxicologie du Québec (Quebec, Canada). Microwave-assisted acid digestion quality control was tested using a freeze-dried biological tissue standard reference material purchased from The National Institute of Standards and Technology (NIST, SRM 2796). Furthermore, previous work by our group evaluated the digestion efficiency of CuO NPs using the above method to be 100% [13]. Performance data on LAMP, QMEQAS, and NIST analyses is provided in the supplemental materials (Additional file 7: Table S5).

### Hemoglobin and ceruloplasmin in serum samples

Whole blood collected by cardiac puncture was incubated at room temperature in a non-heparinized tube for at least 30 min and centrifuged at 2500 × *g* for 10 min to separate serum. Ceruloplasmin, an α-2-glycoprotein containing 95% of the Cu in plasma, functions as a major Cu transporter protein and multi-Cu oxidase protein [55]. Since ceruloplasmin in serum might be correlated to Cu concentrations, we determined these levels using a Ceruloplasmin Colorimetric Activity Kit (Invitrogen, Waltham, MA). The hemoglobin levels were measured using a hemoglobin assay kit (Chondrex Inc., Woodinville, WA), which correlates with a cyanmethemoglobin method, a standard hemoglobin assay.

### Percentage body weight change and dry organ weight change

Percentage body weight changes were calculated from initial body weight (before exposure) subtracted with the weight after exposure. All dry organs were weighed using an analytical balance (Mettler Toledo, Mo. XPR206CDR). Relative organ weights (organ weight to body weight ratio) were presented for liver, kidney, heart, and spleen as the weight of these organs were associated with body weight change. In contrast, brain and lung were presented as the absolute organ weight as their weights were generally not affected by body weight [74, 75].

### Dissolution study of CuO NPs

Relevant dissolution studies of CuO NPs were conducted in three different simulated biological fluids including ALF (pH 4.5), SELF (pH 7.4), and SGF (pH 1.5). The ALF fluid simulated the composition and pH of lysosomes in alveolar and interstitial macrophages while the SELF mimicked interstitial fluid in the lungs. Dissolution in SGF was performed to mimic bioavailability of Cu ions in the gastrointestinal tract since some portion of the NPs might be cleared out through mucociliary escalator activity and swallowed. The composition of SGF, ALF, and SELF can be found in the literature [76–78]. The list of chemicals required to prepare 500 mL 2x solution of ALF, SELF, and SGF are given in Additional file 8: Table S6. Simulated physiological fluids were prepared as stated in references. To prepare SELF, the chemical reagents specified in the inorganic (part A) and organic phase (part B) as shown in Additional file 1: Table S6 were dissolved in 250 mL ultrapure water separately. The order of mixing followed the order listed in Table to prevent precipitation of salt. Prepared inorganic and organic phase solution were combined to form 500 mL solution in a 1-L bottle which contained the additional reagents (part C). The resulting fluid was mixed thoroughly and HCl was added to attain the desired pH of 7.4 ± 0.2. SGF was prepared by dissolving 30 g of glycine into 400 mL ultrapure water. The pH of solution was adjusted to 1.5 with 1 N HCl and then ultrapure water was added until the total volume reached 500 mL. Similar to ALF, all components described in Additional file 1: Table S6 were dissolved in 400 mL of ultrapure water, and the pH of solution was adjusted to 4.5 with 1 N HCl. Then ultrapure water was added to reach 500 mL in the total volume.

CuO NPs were dispersed at 2.0 mg/mL in 16 mL of ultrapure water. The CuO NP suspension was sonicated for the total of 1 min with the cycle of 30 s vortex and then 30 s sonication with the power 220 ~ 230 W. One mL CuO NPs suspension was added to 1 mL 2 × stock simulated fluids (ALF, SELF, or SGF) to a final CuO NP concentration of 1 mg/mL. The samples were incubated on an orbital shaker at 37 °C, 250 rpm continuously for 4 h, 24 h, and 336 h (14 days) (SGF conditions were performed for only 4 and 24 h). The samples were centrifuged for 15 min at 18,800 x* g*, then the supernatant was transferred to Beckman ultracentrifuge tubes and centrifuged for 15 min at 194,400 *x g* (Beckman Coulter, Optima™ XPN-100) to separate undissolved NPs out of the solution. The supernatant was collected and diluted with 2% HNO_3_ at 1:100 dilution for SELF samples, and 1:10,000 dilution for SGF and ALF samples. Samples were analyzed for Cu ions using ICP-MS (Agilent 7900, Santa Clara, CA).

### Statistical analysis

The data were expressed as the mean ± standard deviation (SD). GraphPad Prism (GraphPad Software version 9, San Diego, CA) was used for statistical analysis. Unpaired two-tailed Student’s t-tests were performed when two groups were compared. One-way analysis of variance (ANOVA) followed by Dunnett’s post hoc test was used when more than two groups were compared. If data were not normally distributed, Kruskal–Wallis test was used. Significant differences were considered at p < 0.05. Statistical probability (p values) in plots is expressed as follows: ****p < 0.0001, ***p < 0.001, **p < 0.01, and *p < 0.05.

## Supplementary Information

**Additional file 1**.** Figure S1**. Percentage of Inflammatory cell numbers in BAL fluid of mice exposed to CuO NPs in aerosols: (a) Neutrophils, (b) Macrophages, (c) Lymphocytes, and (d) Eosinophils at different time points throughout or after exposure (Red-highlighted area indicates time during CuO exposure). Statistical analysis for % Neutrophil, % Lymphocyte, and % Macrophage were performed using one-way ANOVA with Dunnett’s post hoc test, while % Eosinophils was performed by Kruskal-Wallis test. Data are expressed as mean ± SD (n = 5). **** P < 0.0001, **P < 0.01,*P < 0.05.

**Additional file 2**.** Table S1**. Total Cu in the lung (μg/mouse lung), total soluble Cu in the lung (μg/mouse lung), and % soluble Cu/ total Cu in the lung.

**Additional file 3**.** Table S2**. Mean dried organ weights and body weights.

**Additional file 4**.** Table S3**. Cu concentration and specific gravity in urine samples.

**Additional file 5**.** Figure S2**. Dissolution of CuO NPs in simulated biological fluids: Simulated gastric fluid (SGF, pH 1.5), artificial lysosomal fluid (ALF, pH 4.5), and simulated epithelial lung fluid (SELF, pH 7.4), n = 2. Percent dissolved Cu was calculated from dissolved Cu over total amount of CuO NPs.

**Additional file 6**.** Table S4**. Reagents, sample composition, and calibrator concentrations for dosimetry analysis using ICP-MS.

**Additional file 7**.** Table S5**. Performance data on NIST, LAMP, and QMEQAS analyses.

**Additional file 8**.** Table S6**. Composition of simulated fluid including ALF (pH = 4.5), SELF (pH = 7.4), and SGF (pH = 1.5) for 500 mL solution preparation for each fluid.

## Data Availability

The datasets used and/or analyzed during the current study are available from the corresponding author on reasonable request.

## Abbreviations

ALF: Artificial lysosomal fluid
ANOVA: One-way analysis of variance
APDC: Ammonium pyrrolidine dithiocarbamate
ARDS: Acute respiratory distress syndrome
BAL: Bronchoalveolar lavage
Bi: Bismuth
Ca: Calcium
Cu: Copper
CuO NPs: Copper oxide nanoparticles
DI: Deionized
DMT: Divalent metal transporter
EDTA: Ethylenediaminetetraacetic acid
Fe: Iron
G-CSF: Granulocyte colony-stimulating factor
GM: Geometric mean
GM-CSF: Granulocyte–macrophage colony-stimulating factor
GSD: Geometric standard deviation
H&E: Hematoxylin and eosin
HNO_3_: Nitric acid
ICP-MS: Inductively coupled plasma-mass spectrometer
IFN: Interferon
Ir: Iridium
K: Potassium
KC: Keratinocyte chemoattractant
LDH: Lactate dehydrogenase
LLOD: Lowest limit of detection
MCP: Monocyte chemotactic protein
MIP: Macrophage inflammatory protein
Mg: Magnesium
Mn: Manganese
MPPD: Multiple path particle dosimetry
PI: Propidium iodide
RBC: Red blood cell
Rh: Rhodium
ROS: Reactive oxygen species
Se: Selenium
SELF: Simulated epithelial lung fluid
SGF: Simulated gastric fluid
SMPS: Scanning mobility particles sizer
Te: Tellurium
TMAH: Tetramethylammonium hydroxide
TNF: Tumor necrosis factor
TWA: Time-weighted average
T_1/2_: Half-life
Y: Yttrium
Zn: Zinc
