# Supplementary material for: Time course of pulmonary inflammation and trace element biodistribution during and after sub-acute inhalation exposure to copper oxide nanoparticles in a murine model
Source: Part Fibre Toxicol. 2022 Jun 13;19:40. doi: 10.1186/s12989-022-00480-z (PMC9195454; doi:10.1186/s12989-022-00480-z)

Figure S1. Percentage of Inflammatory cell numbers in BAL fluid of mice exposed to CuO NPs in aerosols: (a) Neutrophils, (b) Macrophages, (c) Lymphocytes, and (d) Eosinophils at different time points throughout or after exposure (Red-highlighted area indicates time during CuO exposure). Statistical analysis for % Neutrophil, % Lymphocyte, and % Macrophage were performed using one-way ANOVA with Dunnett’s post hoc test, while % Eosinophils was performed by Kruskal-Wallis test. Data are expressed as mean ± SD (n=5). **** P<0.0001, **P<0.01, *P<0.05.


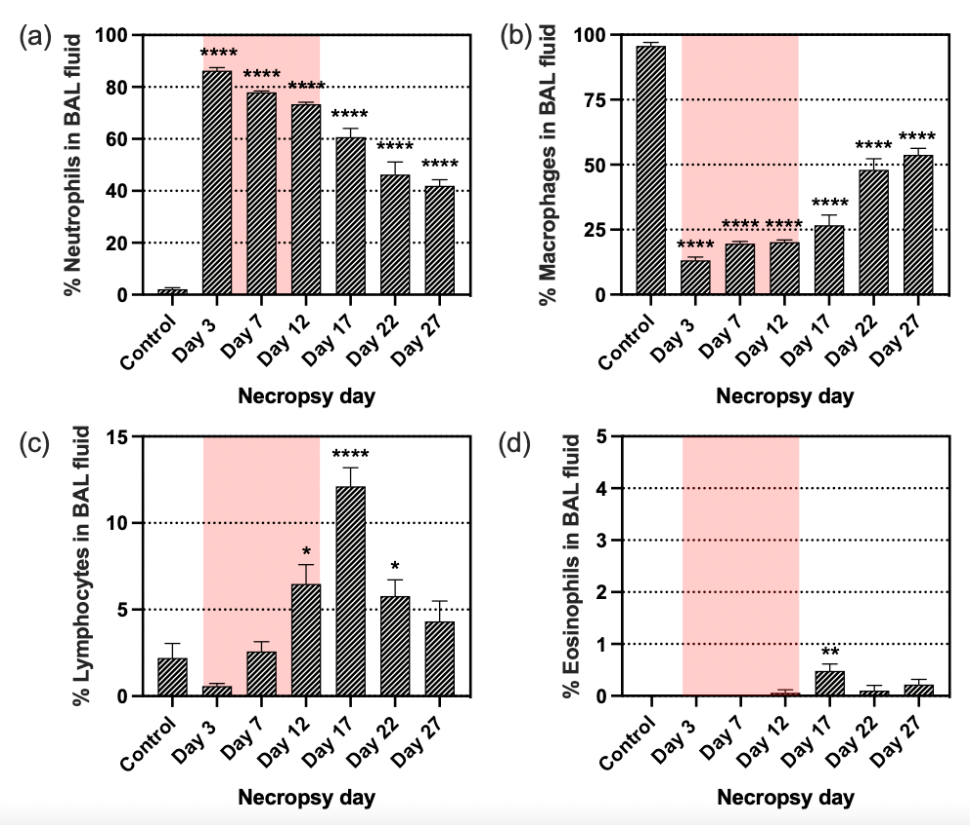

Supplement: Supplementary file 1 — Additional file 1. Figure S1. Percentage of Inflammatory cell numbers in BAL fluid of mice exposed to CuO NPs in aerosols: (a) Neutrophils, (b) Macrophages, (c) Lymphocytes, and (d) Eosinophils at different time points throughout or after exposure (Red-highlighted area indicates time during CuO exposure). Statistical analysis for % Neutrophil, % Lymphocyte, and % Macrophage were performed using one-way ANOVA with Dunnett’s post hoc test, while % Eosinophils was performed by Kruskal-Wallis test. Data are expressed as mean ± SD (n = 5). **** P < 0.0001, **P < 0.01,*P < 0.05. [file 12989_2022_480_MOESM1_ESM.docx]
