# Supplementary material for: Time course of pulmonary inflammation and trace element biodistribution during and after sub-acute inhalation exposure to copper oxide nanoparticles in a murine model
Source: Part Fibre Toxicol. 2022 Jun 13;19:40. doi: 10.1186/s12989-022-00480-z (PMC9195454; doi:10.1186/s12989-022-00480-z)
Supplement: Supplementary file 2 — Additional file 2. Table S1. Total Cu in the lung (μg/mouse lung), total soluble Cu in the lung (μg/mouse lung), and % soluble Cu/ total Cu in the lung. [file 12989_2022_480_MOESM2_ESM.docx]

Table S1. Total Cu in the lung (μg/mouse lung), total soluble Cu in the lung (μg/mouse lung), and % soluble Cu/ total Cu in the lung.

| Experimental groups | Total Cu (μg/mouse lung) | Total soluble Cu  (μg/mouse lung) | % Soluble Cu/ Total Cu in the lung |
| --- | --- | --- | --- |
|  | mean (SD) | mean (SD) | mean (SD) |
| Control | 0.392 (0.051) | 0.018 (0.004) | 4.69 (1.01) |
| Day 3 | 5.68 (0.63) | 0.085 (0.032) | 1.52 (0.62) |
| Day 7 | 5.67 (0.58) | 0.140 (0.014) | 2.50 (0.47) |
| Day 12 | 10.15 (1.02) | 0.204 (0.042) | 2.02 (0.43) |
| Day 17 | 5.90 (0.41) | 0.153 (0.046) | 2.57 (0.68) |
| Day 22 | 4.06 (0.42) | 0.088 (0.012) | 2.18 (0.32) |
| Day 27 | 2.50 (0.51) | 0.055 (0.007) | 2.26 (0.41) |
