# Supplementary material for: Time course of pulmonary inflammation and trace element biodistribution during and after sub-acute inhalation exposure to copper oxide nanoparticles in a murine model
Source: Part Fibre Toxicol. 2022 Jun 13;19:40. doi: 10.1186/s12989-022-00480-z (PMC9195454; doi:10.1186/s12989-022-00480-z)
Supplement: Supplementary file 3 — Additional file 3. Table S2. Mean dried organ weights and body weights. [file 12989_2022_480_MOESM3_ESM.docx]

Table S2. Mean dried organ weights and body weights.

| Experimental groups | Mean dried organ weight, mg (SD) | | | | | | Mean body weight, g (SD) |
| --- | --- | --- | --- | --- | --- | --- | --- |
|  |  | | | | | |  |
|  | Liver | Kidney | Heart | Spleen | Brain | Lung |  |
| Control | 343.5 (47.4) | 69.9 (6.2) | 27.2 (1.4) | 20.7  (3.2) | 97.5 (5.2) | 10.6  (1.2) | 23.4  (2.7) |
| Day 3 | 254.7 (16.9) | 77.5 (8.3) | 23.7 (1.5) | 13.9  (1.1) | 87.2 (5.5) | 10.2  (0.9) | 20.6  (0.9) |
| Day 7 | 261.0 (15.8) | 63.1 (3.2) | 23.5 (1.9) | 14.1  (1.0) | 92.9 (4.7) | 11.3  (0.3) | 20.3  (0.8) |
| Day 12 | 254.6 (7.2) | 66.7 (5.7) | 23.1 (1.9) | 12.7  (0.4) | 94.4 (4.6) | 12.2  (0.8) | 20.5  (0.6) |
| Day 17 | 286.0 (29.0) | 73.2 (8.6) | 23.7 (2.0) | 15.7  (1.5) | 96.3 (6.6) | 13.0  (1.0) | 19.8  (1.0) |
| Day 22 | 312.8 (26.4) | 72.1 (3.6) | 26.0 (2.9) | 18.9  (0.8) | 96.1 (7.2) | 13.0  (0.7) | 20.4  (1.6) |
| Day 27 | 320.8 (12.4) | 71.5 (4.8) | 26.4 (1.5) | 17.6  (1.8) | 98.0 (3.7) | 12.9  (0.7) | 20.9  (0.5) |
