# Supplementary material for: Time course of pulmonary inflammation and trace element biodistribution during and after sub-acute inhalation exposure to copper oxide nanoparticles in a murine model
Source: Part Fibre Toxicol. 2022 Jun 13;19:40. doi: 10.1186/s12989-022-00480-z (PMC9195454; doi:10.1186/s12989-022-00480-z)
Supplement: Supplementary file 4 — Additional file 4. Table S3. Cu concentration and specific gravity in urine samples. [file 12989_2022_480_MOESM4_ESM.docx]

Table S3. Cu concentration and specific gravity in urine samples.

| Experimental groups | Cu concentration (ug/L) | Specific gravity  (Sp Gr, g/mL) | Normalized factor | Sp Gr normalized Cu concentration (ug/L) |
| --- | --- | --- | --- | --- |
| Control | 123.094 | 1.025 | 1.000 | 123.076 |
| Day3 | 266.361 | 1.083 | 1.056 | 252.191 |
| Day7 | 178.856 | 1.058 | 1.032 | 173.352 |
| Day12 | 190.727 | 1.005 | 0.980 | 194.532 |
| Day17 | 132.069 | 1.006 | 0.982 | 134.523 |
| Day 22 | 92.270 | 1.002 | 0.977 | 94.439 |
| Day 27 | 104.101 | 0.997 | 0.973 | 107.025 |
