# Supplementary material for: Time course of pulmonary inflammation and trace element biodistribution during and after sub-acute inhalation exposure to copper oxide nanoparticles in a murine model
Source: Part Fibre Toxicol. 2022 Jun 13;19:40. doi: 10.1186/s12989-022-00480-z (PMC9195454; doi:10.1186/s12989-022-00480-z)
Supplement: Supplementary file 5 — Additional file 5. Figure S2. Dissolution of CuO NPs in simulated biological fluids: Simulated gastric fluid (SGF, pH 1.5), artificial lysosomal fluid (ALF, pH 4.5), and simulated epithelial lung fluid (SELF, pH 7.4), n = 2. Percent dissolved Cu was calculated from dissolved Cu over total amount of CuO NPs. [file 12989_2022_480_MOESM5_ESM.docx]

Figure S2. Dissolution of CuO NPs in simulated biological fluids: Simulated gastric fluid (SGF, pH 1.5), artificial lysosomal fluid (ALF, pH 4.5), and simulated epithelial lung fluid (SELF, pH 7.4), n=2. Percent dissolved Cu was calculated from dissolved Cu over total amount of CuO NPs.
