# Supplementary material for: Time course of pulmonary inflammation and trace element biodistribution during and after sub-acute inhalation exposure to copper oxide nanoparticles in a murine model
Source: Part Fibre Toxicol. 2022 Jun 13;19:40. doi: 10.1186/s12989-022-00480-z (PMC9195454; doi:10.1186/s12989-022-00480-z)
Supplement: Supplementary file 6 — Additional file 6. Table S4. Reagents, sample composition, and calibrator concentrations for dosimetry analysis using ICP-MS. [file 12989_2022_480_MOESM6_ESM.docx]

Table S4. Reagents, sample composition, and calibrator concentrations for dosimetry analysis using ICP-MS.

| Whole blood sample | |
| --- | --- |
| - Diluent | 0.4% v/v tetramethylammonium hydroxide (TMAH), 1% v/v ethanol, 0.01% w/v ammonium pyrrolidine dithiocarbamate (APDC), 0.05% v/v triton X-100, and 5 μg/L of each iridium, rhodium, tellurium (internal standard) in DI water |
| - Rinse solution | 0.4% v/v TMAH, 1% v/v ethanol, 0.01% w/v APDC, and 0.05% v/v triton X-100 in DI water |
| - Sample preparation | 100 μL whole blood sample + 100 μL DI water + 4800 μL diluent |
| - Matrix blank and calibrators (S0-S5) | 100 μL base blood + 100 μL Trace (S0-S5) + 4800 μL diluent |
| - Reagent blank | 200 μL DI water + 4800 μL diluent |
| - Quality control standard | 100 μL QM-B-Q1821 + 100 μL DI water + 4800 μL diluent |
| Urine sample | |
| - Diluent | 10 µg/L Rh in 2% v/v HNO_3_ + 1.5% v/v ethanol |
| - Rinse solution | 0.002% triton-x100 + 2% v/v HNO_3_ + 1.5% v/v ethanol solution |
| - Sample preparation | 100 μL urine sample + 100 μL DI water + 1800 μL diluent |
| - Matrix blank and calibrators (S0-S5) | 540 μL base urine + 60 μL trace (S0-S5) + 5400 μL diluent |
| - Reagent blank | 200 μL DI water + 4800 μL diluent |
| - Quality control standard | 100 μL QM-U-Q1906 + 100 μL DI water + 1800 μL diluent |
