# Supplementary material for: Time course of pulmonary inflammation and trace element biodistribution during and after sub-acute inhalation exposure to copper oxide nanoparticles in a murine model
Source: Part Fibre Toxicol. 2022 Jun 13;19:40. doi: 10.1186/s12989-022-00480-z (PMC9195454; doi:10.1186/s12989-022-00480-z)
Supplement: Supplementary file 7 — Additional file 7. Table S5. Performance data on NIST, LAMP, and QMEQAS analyses. [file 12989_2022_480_MOESM7_ESM.docx]

Table S5. Performance data on NIST, LAMP, and QMEQAS analyses.

| Element | Sample ID | Sample matrix | Unit | Average | n | 1 SD | Target | % Recovery |
| --- | --- | --- | --- | --- | --- | --- | --- | --- |
| Ca | NIST SRM 2796 | Freeze dried tissue | µg/g dry wt tissue | 6940 | 3 | 414 | 7600 | 91% |
| Mn | NIST SRM 2796 | Freeze dried tissue | µg/g dry wt tissue | 34 | 3 | 0.15 | 33 | 103% |
| Fe | NIST SRM 2796 | Freeze dried tissue | µg/g dry wt tissue | 154 | 3 | 2.4 | 171 | 90% |
| Cu | NIST SRM 2796 | Freeze dried tissue | µg/g dry wt tissue | 3.6 | 3 | 0.14 | 4 | 90% |
| Zn | NIST SRM 2796 | Freeze dried tissue | µg/g dry wt tissue | 139 | 3 | 14.4 | 137 | 101% |
| Se | NIST SRM 2796 | Freeze dried tissue | µg/g dry wt tissue | 1.7 | 3 | 0.21 | 1.8 | 94% |
| Mn | LAMP 1904 | Whole blood | µg/L | 8.2 | 4 | 0.9 | 7.4 | 111% |
|  | LAMP 1905 | Whole blood | µg/L | 13.4 | 4 | 1.1 | 12.8 | 105% |
|  | LAMP 1906 | Whole blood | µg/L | 8.4 | 4 | 0.8 | 7.8 | 108% |
|  | LAMP 1907 | Whole blood | µg/L | 15.3 | 4 | 2.0 | 13.2 | 116% |
|  | LAMP 1908 | Whole blood | µg/L | 10.7 | 4 | 2.1 | 8.3 | 129% |
|  | LAMP 1909 | Whole blood | µg/L | 11.2 | 4 | 1.1 | 9.6 | 117% |
|  | LAMP 1910 | Whole blood | µg/L | 22.2 | 4 | 0.4 | 23.1 | 96% |
|  | LAMP 1911 | Whole blood | µg/L | 13.1 | 4 | 0.3 | 12.7 | 103% |
|  | LAMP 1912 | Whole blood | µg/L | 6.1 | 4 | 0.1 | 5.4 | 113% |
|  | LAMP 2001 | Whole blood | µg/L | 9.3 | 4 | 0.1 | 9.1 | 102% |
|  | LAMP 2002 | Whole blood | µg/L | 5.8 | 4 | 0.4 | 6.2 | 94% |
|  | LAMP 2003 | Whole blood | µg/L | 3.4 | 4 | 0.2 | 3.5 | 97% |
| Se | LAMP 1904 | Whole blood | µg/L | 212.8 | 4 | 15.4 | 218.9 | 97% |
|  | LAMP 1905 | Whole blood | µg/L | 200.3 | 4 | 11.6 | 206.4 | 97% |
|  | LAMP 1906 | Whole blood | µg/L | 212.7 | 4 | 15.6 | 221.2 | 96% |
|  | LAMP 1907 | Whole blood | µg/L | 201.2 | 4 | 10.7 | 216.5 | 93% |
|  | LAMP 1908 | Whole blood | µg/L | 243.6 | 4 | 12.9 | 236.3 | 103% |
|  | LAMP 1909 | Whole blood | µg/L | 201.3 | 4 | 12.2 | 229.4 | 88% |
|  | LAMP 1910 | Whole blood | µg/L | 237 | 4 | 6.7 | 246.4 | 96% |
|  | LAMP 1911 | Whole blood | µg/L | 225 | 4 | 4.6 | 227.2 | 99% |
|  | LAMP 1912 | Whole blood | µg/L | 169.3 | 4 | 10.3 | 166.3 | 102% |
|  | LAMP 2001 | Whole blood | µg/L | 188.8 | 4 | 7.2 | 216.1 | 87% |
|  | LAMP 2002 | Whole blood | µg/L | 185.9 | 4 | 4.6 | 215 | 86% |
|  | LAMP 2003 | Whole blood | µg/L | 155.6 | 4 | 6.7 | 177.9 | 87% |
| Cu | QM-B-Q1821 | Whole blood | µg/L | 1944.8 | 2 | 107.0 | 1638 | 119% |
| Mn | QM-B-Q1821 | Whole blood | µg/L | 12.5 | 2 | 1.5 | 13 | 96.15% |
| Zn | QM-B-Q1821 | Whole blood | µg/L | 9525.5 | 2 | 220.7 | 8128 | 117.19% |
| Se | QM-B-Q1821 | Whole blood | µg/L | 338.1 | 2 | 25.6 | 335.4 | 100.81% |
| Cu | QM-U-Q1906 | Urine | µg/L | 19.4 | 1 | N/A | 22 | 88.18% |
