# Supplementary material for: Time course of pulmonary inflammation and trace element biodistribution during and after sub-acute inhalation exposure to copper oxide nanoparticles in a murine model
Source: Part Fibre Toxicol. 2022 Jun 13;19:40. doi: 10.1186/s12989-022-00480-z (PMC9195454; doi:10.1186/s12989-022-00480-z)
Supplement: Supplementary file 8 — Additional file 8. Table S6. Composition of simulated fluid including ALF (pH = 4.5), SELF (pH = 7.4), and SGF (pH = 1.5) for 500 mL solution preparation for each fluid. [file 12989_2022_480_MOESM8_ESM.docx]

Table S6. Composition of simulated fluid including ALF (pH=4.5), SELF (pH=7.4), and SGF (pH=1.5).

| ALF (pH=4.5) | | SELF (pH=7.4) | | | SGF (pH=1.5) | |
| --- | --- | --- | --- | --- | --- | --- |
| Reagents | Amount (g)* | Reagents | | Amount (g)* | Reagents | Amount (g)* |
| NaCl | 3.21 | (A) Inorganic phase reagents | NaCl | 6.02 |  | |
| CaCl_2_.2H_2_0 | 0.128 |  | CaCl_2_ | 0.256 |  |  |
| Na_2_HPO_4_ | 0.071 |  | Na_2_HPO_4_ | 0.15 |  |  |
|  | |  | NaHCO_3_ | 2.7 |  |  |
|  |  |  | KCl | 0.298 |  |  |
| MgCl_2_ | 0.05 |  | MgCl_2_ | 0.2 |  |  |
| Na_2_SO_4_ | 0.039 |  | Na_2_SO_4_ | 0.072 |  |  |
| C_6_H_5_Na_3_O_7_.2H_2_O | 0.077 | (B) Organic phase reagents | Ascorbic acid | 0.018 |  |  |
| NaOH | 6 |  | Uric acid | 0.016 |  |  |
|  | |  | Glutathione | 0.03 |  |  |
| C_6_H_8_O_7_ | 20.8 | (C) Additional reagents | Albumin | 0.26 |  |  |
| C_3_H_5_NaO_3_ | 0.085 |  | Cysteine | 0.122 |  |  |
| C_4_H_4_O_6_Na_2_.2H_2_O | 0.09 |  | DPPC | 0.1 |  |  |
| Glycine | 0.059 |  | Glycine | 0.376 | Glycine | 30 |
| C_3_H_3_O_3_Na | 0.086 |  | Mucin | 0.5 |  | |

* Weight of reagents for 500 mL 2x solution preparation
